# Context-dependent dysregulation of store-operated calcium channels in head and neck squamous cell carcinoma

**DOI:** 10.1371/journal.pone.0344393

**Published:** 2026-03-09

**Authors:** Heba Ghozlan, Saja Al-Malahmeh, Othman Al-Shboul, Anas J. Mistareehi, Lina Elsalem

**Affiliations:** 1 Department of Physiology and Biochemistry, Faculty of Medicine, Jordan University of Science and Technology, Irbid, Jordan; 2 Department of Anatomy, Faculty of Medicine, Jordan University of Science and Technology, Irbid, Jordan; 3 Department of Pharmacology, Faculty of Medicine, Jordan University of Science and Technology, Irbid, Jordan; King Faisal Specialist Hospital and Research Center, SAUDI ARABIA

## Abstract

Store-operated calcium entry (SOCE), mediated by ORAI1–3 calcium channels and stromal interaction molecules STIM1 and STIM2, is increasingly recognized as a regulator of cancer progression. However, its role in head and neck squamous cell carcinoma (HNSCC) and its relationship with major oncogenic pathways remain poorly defined. Transcriptomic and clinical data from The Cancer Genome Atlas (TCGA) were analyzed to profile isoform-specific ORAI1–3 and STIM1–2 expression across HNSCC subtypes and oncogenic contexts. In parallel, the effects of pharmacologic SOCE inhibition with 2-aminoethoxydiphenyl borate (2-APB) were evaluated in FaDu (epidermal growth factor receptor [EGFR]-high, PIK3CA–wild-type) and Detroit-562 (metastatic, PIK3CA–mutant) cells by assessing viability, migration, and clonogenic survival. TCGA analysis revealed a context-dependent SOCE expression profile. ORAI1–3 and STIM2 were broadly upregulated in tumors, while STIM1 was significantly downregulated, particularly in advanced and basaloid subtypes. PIK3CA mutations, especially the H1047R hotspot, were associated with higher STIM1 expression, whereas EGFR expression correlated positively with STIM1/2 but negatively with ORAI1/3. In vitro, Detroit-562 cells expressed higher levels of SOCE components and showed greater sensitivity to SOCE inhibition, with marked reductions in viability, migration, and clonogenic capacity. FaDu cells, despite higher EGFR expression, exhibited lower SOCE gene expression and relative resistance to 2-APB, which suggests reduced dependence on SOCE-mediated signaling. These findings suggest that SOCE components are transcriptionally dysregulated in HNSCC and may represent a context-dependent therapeutic vulnerability, particularly in PIK3CA-mutant tumors. Validation in additional preclinical models, patient-derived xenografts, and clinical specimens is required to establish SOCE as a biomarker and therapeutic target in HNSCC.

## Introduction

Head and neck squamous cell carcinoma (HNSCC) encompasses tumors of the oral cavity, nasopharynx, oropharynx, hypopharynx, and larynx, with considerable variation in etiology, epidemiology, and treatment response [1]. Major risk factors include tobacco and alcohol consumption as well as high-risk human papillomavirus (HPV) infection [1]. Globally, HNSCC accounts for over 890,000 new cases and 450,000 deaths each year, with marked geographical and sex-related disparities in incidence and outcome [2]. Despite therapeutic advances, prognosis remains poor, particularly for patients diagnosed with advanced-stage disease [3]. The molecular heterogeneity of HNSCC, driven by recurrent TP53 mutations, frequent EGFR amplification or overexpression, and activating PIK3CA mutations, underpins its adaptability and resistance to therapy [4–8]. These oncogenic pathways are tightly interconnected, with RAS signaling functioning as a critical upstream regulator of the PI3K/AKT axis in HNSCC [9]. This complex signaling network highlights the importance of defining how major oncogenic drivers rewire core cellular processes, such as calcium signaling, to sustain tumor growth and progression.

Calcium (Ca^2+^) signaling plays a central role in cancer biology as a universal second messenger controlling proliferation, migration, invasion, and resistance to stress [10,11]. Store-operated calcium entry (SOCE), the principal Ca^2+^ influx pathway in non-excitable cells, is mediated by stromal interaction molecules (STIM1 and STIM2), which sense endoplasmic reticulum Ca^2+^ depletion and activate ORAI channels (ORAI1, ORAI2, and ORAI3), which form the pore at the plasma membrane [11–14]. STIM1 and STIM2 differ in calcium-sensing thresholds and activation strength. STIM2 responds to subtle ER depletion and sustains basal calcium influx [15,16], while STIM1, the canonical activator of SOCE, requires robust depletion and drives strong, receptor-activated SOCE [17–19]. Similarly, ORAI1 is essential for robust store-operated currents [20,21], whereas ORAI2 and ORAI3 form heteromeric complexes that fine-tune signaling and confer resistance to oxidative stress [22–24]. These isoforms can form homomeric or heteromeric channels, which allow cells to fine-tune calcium influx under different physiological or stress conditions. This isoform diversity may contribute variably to optimize calcium signaling for distinct malignant behaviors.

Dysregulated SOCE has been implicated in multiple cancers, where abnormal ORAI/STIM activity promotes proliferation, invasion, metastasis, and therapeutic resistance [25]. In HNSCC, elevated ORAI1 and STIM1 expression has been reported in advanced tumors, which correlates with lymph node metastasis and pathologic stage [26]. Pharmacologic or genetic inhibition of ORAI1/2 suppresses tumorigenesis, which supports their oncogenic role [27]. Recent single-cell RNA analyses revealed differential ORAI and STIM isoform expression that correlated with mitochondrial dynamics, but the biological significance of these patterns remains unexplored [28]. Collectively, these findings indicate that SOCE contributes to HNSCC progression. However, most studies have focused on individual isoforms, without examining coordinated ORAI and STIM expression, stage-specific alterations, or interactions with major oncogenic drivers such as PIK3CA mutations or EGFR overexpression. Moreover, although genetic knockdown experiments demonstrate the oncogenic potential of specific SOCE components [26,27,29], the functional consequences of pharmacologic SOCE inhibition in molecularly defined HNSCC models remain incompletely characterized. The role of SOCE in primary versus metastatic disease, a key aspect of tumor progression, has also not been systematically addressed.

To address these gaps, we integrated transcriptomic and clinical data from The Cancer Genome Atlas (TCGA) to characterize isoform-specific dysregulation of ORAI and STIM genes across HNSCC subtypes and oncogenic contexts, including PIK3CA mutation and EGFR expression. We then assessed the functional impact of SOCE inhibition using 2-aminoethoxydiphenyl borate (2-APB) in two representative HNSCC cell lines: FaDu (primary hypopharyngeal, EGFR-high, PIK3CA-wild-type) and Detroit-562 (metastatic pharyngeal, PIK3CA H1047R-mutant). While 2-APB is not fully isoform-specific, it allows rapid, reversible, and dose-titratable inhibition of ORAI-mediated calcium entry that provides a tractable approach to functionally probe SOCE function in HNSCC. By combining in silico and in vitro analyses, this study evaluates SOCE as a potential therapeutic vulnerability, particularly in advanced and metastatic disease.

## Materials and methods

### Ethics statement

This study utilized publicly available transcriptomic and clinical data from The Cancer Genome Atlas (TCGA), which were originally accessed on 3 September 2024 via UCSC Xena and cBioPortal, and re-queried on 2 August 2025 for figure generation and validation. All patient data were fully de-identified and accessible in accordance with TCGA data sharing policies and ethical standards; authors did not have access to any identifiable participant information. Human cell lines (FaDu and Detroit-562) used in functional assays were obtained from authenticated commercial sources (American Type Culture Collection, ATCC). All cell culture and experimental procedures complied with institutional biosafety and ethical regulations. No new human participants or animal subjects were involved in this study; therefore, no additional institutional review board (IRB) approval or informed consent was required.

### Bioinformatics analysis

Publicly available data were analyzed using the UCSC Xena browser (University of California, Santa Cruz, CA, USA; https://xena.ucsc.edu/) and cBioPortal for Cancer Genomics (https://www.cbioportal.org/). The TCGA-TARGET-GTEx integrated dataset, which includes The Cancer Genome Atlas (TCGA), Therapeutically Applicable Research to Generate Effective Treatments (TARGET), and Genotype-Tissue Expression (GTEx) projects, was analyzed for ORAI1–3 and STIM1–2 mRNA expression in HNSCC versus normal tissues. Somatic mutation profiles, genomic alteration frequencies, and correlation analyses were assessed using the TCGA PanCancer Atlas HNSCC cohort via cBioPortal. Parallel analysis of copy number variations and expression profiles was conducted using the Cancer Cell Line Encyclopedia (CCLE) database. Data visualization was generated using the default UCSC Xena parameters, which include boxplots for tissue comparisons and heatmaps for cell line analysis.

### Cell lines and culture

The HNSCC cell lines FaDu and Detroit-562 were obtained from the American Type Culture Collection (ATCC, Manassas, VA, USA). FaDu is a human hypopharyngeal squamous cell carcinoma (HNSCC) cell line derived from a hypopharyngeal tumor, while Detroit-562 is a human pharyngeal carcinoma cell line established from a metastatic pharyngeal carcinoma to the pleural fluid. Cells were cultured in Minimum Essential Medium (MEM; Euroclone S.P.A, Pero, Italy) supplemented with 10% fetal bovine serum (FBS; Cytiva, Vienna, Austria), 1% penicillin–streptomycin (Euroclone S.P.A, Pero, Italy), 1% L-glutamine (Euroclone S.P.A, Pero, Italy), and 1% non-essential amino acids (Euroclone, Italy). Cultures were maintained at 37 °C and 5% CO₂. Cells were routinely passaged at 80–90% confluence to maintain exponential growth.

### RNA isolation and quantitative PCR

#### Primer design and validation.

All primers were designed using Primer3 (v.2.5.0) with design criteria optimized for qPCR applications: [1] product size 80–240 bp; [2] melting temperature (Tm) 58–62°C; [3] GC content 45–65%; [4] minimal self-complementarity and hairpin formation potential. Primer specificity was validated in silico using NCBI Primer–BLAST (https://www.ncbi.nlm.nih.gov/tools/primer-blast/) against the human RefSeq mRNA database and genome assembly (GRCh38/hg38). Additional validation was performed using UCSC InSilico PCR (https://genome.ucsc.edu/cgi-bin/hgPcr) against GENCODE transcript annotations (v38) to predict both cDNA-specific and genomic amplicon sizes. Primer sequences and *in silico* validation summaries are provided in Supplementary Tables S1-S2 in S1 File.

#### RNA isolation and cDNA synthesis.

Total RNA was extracted from cell samples using the Direct-zol™ RNA Miniprep Kit (ZYMO Research, California, USA, R2050) according to the manufacturer's protocol. To minimize genomic DNA contamination, all samples underwent on-column DNase I digestion: following RNA binding to the ZymoSpin™ column, 15 μL DNase I solution (supplied with the kit) was applied directly to the column membrane and incubated at room temperature for 15 minutes, followed by two washes with RNA Wash Buffer. RNA quality and concentration were assessed using a NanoDrop 2000 spectrophotometer (Nabi UV/Vis Nano Spectrophotometer, Seongnam, Korea), with A260/A280 ratios of 1.8–2.0 considered acceptable. Complementary DNA (cDNA) was synthesized from 500 ng of total RNA using the 5X PrimeScript RT Master Mix (Takara, Japan, RR036A) according to the manufacturer's instructions.

#### Genomic DNA contamination controls.

To ensure amplification specificity and confirm effective removal of genomic DNA, multiple quality control measures were implemented. First, for each RNA sample, parallel cDNA synthesis reactions were performed with (+RT) and without (-RT) reverse transcriptase using identical RNA input (500 ng). All -RT controls were analyzed by qPCR alongside experimental samples using all primer sets. All -RT controls showed either no amplification (Ct > 35) or large ΔCt values (>10) between +RT and -RT reactions, confirming that genomic DNA contribution was negligible (<0.1% of total signal). Second, each qPCR plate included no-template control (NTC) wells containing nuclease-free water to detect potential primer-dimer formation or reagent contamination; all NTCs showed no amplification. Third, dissociation curve analysis (60–95°C ramp) following each qPCR run revealed single melting peaks for all primer pairs, confirming amplification of specific products without primer-dimers or non-specific amplicons. Representative melt curves are shown in Supplementary Figure S7A-F in S2 File.

#### Quantitative PCR.

Quantitative PCR was performed using TB Green Premix Ex Taq II (Takara, Shiga, Japan, RR820L) with gene-specific primers for ORAI1–3, STIM1–2, and GAPDH (Supplementary Table S1 in S1 File) on a StepOnePlus Real-Time PCR System (Applied Biosystems, California, USA). Reactions were performed in 20 μL volumes containing 10 μL TB Green Premix Ex Taq II (2×), 0.4 μL each primer (10 μM), 2 μL cDNA template, and 7.2 μL nuclease-free water. Thermal cycling conditions consisted of initial denaturation at 95°C for 30 seconds, followed by 40 cycles of 95°C for 5 seconds and 60°C for 30 seconds. Relative gene expression was calculated using the 2 method with GAPDH as the endogenous reference gene. All samples were analyzed in technical duplicates or triplicates, and mean Ct values were used for calculations.

### Pharmacological inhibition

2-Aminoethoxydiphenyl borate (2-APB; Sigma-Aldrich, Darmstadt, Germany, 100065−100MG), a known pharmacological SOCE inhibitor, was dissolved in DMSO (GeneON GmbH, Groß-Rohrheim, Germany) to prepare 100 mM stock solutions and stored at −20°C. Working concentrations were freshly prepared in culture medium immediately before use, ensuring a final DMSO concentration of ≤ 0.1%. 2-APB is a well-characterized pharmacological SOCE inhibitor that directly blocks ORAI channel pore conductance and disrupts STIM-ORAI coupling at ER-plasma membrane junctions [30,31]. 2-APB acts rapidly and reversibly to inhibit store-operated calcium influx without altering ORAI or STIM protein expression levels. Effective SOCE pathway inhibition was confirmed through multiple functional readouts (viability, migration, clonogenic survival) rather than protein-level measurements, consistent with 2-APB's mechanism as a direct channel blocker.

### Cell viability assay

Cells were seeded in 96-well plates at a density of 5,000 cells per well and allowed to adhere overnight. The following day, cells were treated with increasing concentrations of 2-APB for 48 hours to evaluate viability using MTT assay. In brief, thiazolyl blue tetrazolium bromide (Sigma-Aldrich, Darmstadt, Germany, M5655-1G) stock solution (5 mg/mL) was added to each well at 10% of the final volume (10 μL per 100 μL medium) and incubated for 3–4 hours at 37 °C. The medium was then removed, and 50 µL DMSO was added to each well to solubilize the formazan crystals, followed by gentle shaking for 15–20 minutes. Absorbance was measured at 570 nm using a microplate reader (BioTek Synergy H1, Winooski, USA). Cell viability was normalized to DMSO-treated controls using the formula:

% Viability = (Absorbance of sample at 570 nm / Mean absorbance of control at 570 nm) × 100

### Wound healing assay

The effect of 2-APB on HNSCC cell migration was assessed by wound assay. FaDu and Detroit-562 cells were seeded in 12-well plates at densities of 150,000 and 180,000 cells per well, respectively, and cultured until they reached 90–100% confluency. A uniform scratch was then manually created across the monolayer using a 200 μL pipette tip and cells were washed with PBS (Euroclone, Pero, Italy) to remove non-adherent cells. Cultures were maintained in complete MEM containing 5% FBS and treated with either 2-APB or vehicle (DMSO) for 12 hours. FaDu and Detroit-562 cells were treated with 200 µM and 100 µM 2-APB, respectively. Wound closure was imaged at 5X magnification immediately (0 h) and after 12 h using a light microscope (AmScope, MU900, California, USA). The migration rate was calculated using ImageJ software (Version 1.54g, National Institutes of Health, USA) according to the following formula:

Migration rate (%) = ((Distance at 0 h − Distance at 12 h) / Distance at 0 h) × 100

### Colony formation assay

The colony formation assay was performed as previously described [32] with minor modifications. FaDu and Detroit-562 cells were seeded in 12-well plates at a density of 250 cells per well in 1 ml of complete medium. After 3–5 hours, cells were treated with 200 µM (FaDu) or 100 µM (Detroit-562) 2-APB, while control cells received DMSO. Cells were maintained at 37 °C with 5% CO₂. For long-term exposure, cells were continuously treated with 2-APB or DMSO for 3–4 weeks, with medium refreshed every 48–72 hours. For recovery experiments, cells were treated for 72 hours, after which the medium was replaced with drug-free medium and incubated for an additional 3–4 weeks to allow colony formation. Colonies were washed 2–3 times with PBS, fixed with 500 µL absolute methanol (Honeywell, Seelze, Germany) for 20 minutes, and stained with 500 µL of 0.5% crystal violet for 20–40 minutes at room temperature in the dark. Excess stain was removed by washing with PBS and tap water, and the plates were air-dried before imaging. Colony area (%) was quantified using ImageJ software.

### Statistical analysis

Data are presented as mean ± standard deviation (SD) or standard error of the mean (SEM) from at least three independent biological replicates. Statistical comparisons between two groups were performed using Student's t-test, while multiple-group comparisons were assessed by one-way ANOVA followed by appropriate post-hoc tests. All analyses were conducted using GraphPad Prism (version 8; GraphPad Software, San Diego, CA, USA). A *p*-value < 0.05 was considered statistically significant.

## Results

### Isoform-specific dysregulation of ORAI and STIM genes in HNSCC and their clinical correlates

Expression of ORAI and STIM, components of SOCE, is dysregulated in several cancer types. To investigate their role in HNSCC progression, we compared their gene expression patterns in HNSCC tissues vs normal tissues using publicly available cancer databases. Gene expression patterns of ORAI isoforms (ORAI1, ORAI2, and ORAI3) and STIM isoforms (STIM1, STIM2) were evaluated using the UCSC Xena Browser based on the TCGA HNSCC dataset (n = 566 samples).

**Fig 1:**
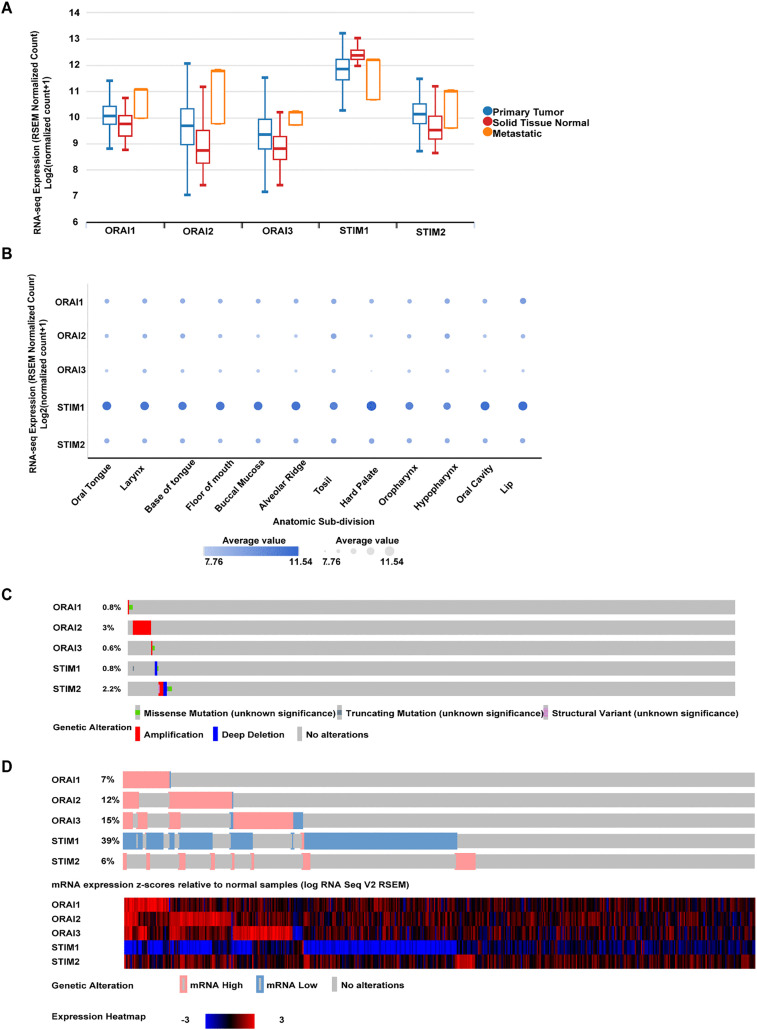
Differential expression and genomic alteration of SOCE components in HNSCC. **(A)** Boxplot representation of mRNA expression levels of ORAI1, ORAI2, ORAI3, STIM1, and STIM2 across normal solid tissue (red), primary tumors (blue), and metastatic tissues (orange). Data were obtained from the UCSC Xena Browser using the TCGA HNSCC cohort (n = 566). Expression is shown as log₂(normalized count + 1). One-way ANOVA revealed significant differences across groups: ORAI1 (p < 0.001), ORAI2 (p < 0.001), ORAI3 (p < 0.01), STIM1 (p < 0.001), and STIM2 (p < 0.001). **(B)** Dot plot of ORAI and STIM isoform expression across HNSCC anatomical subsites in the TCGA cohort (n = 604), t-test (ORAI1: p > 0.05; ORAI2: p < 0.01; ORAI3: p < 0.01; STIM1: p < 0.05; STIM2: p > 0.05). **(C)** OncoPrint visualization of somatic alterations in ORAI/STIM genes in the TCGA PanCancer Atlas HNSCC cohort (n = 523). **(D)** OncoPrint visualization and heatmap of the same cohort showing mRNA alterations relative to normal samples.

mRNA expression profile across normal solid tissues, primary tumors, and metastatic tissues revealed a significant deregulation of SOCE components (Fig 1A). All ORAI isoforms showed increased mRNA expression in primary and metastatic tumor samples relative to normal tissues (ORAI1: p < 0.001; ORAI2: p < 0.001; ORAI3: p < 0.01). Similarly, STIM2 expression was significantly higher in metastatic and primary tumors compared to normal tissues (p < 0.001). In contrast, STIM1 expression was reduced in primary and metastatic tumors compared with normal tissues (p < 0.001).

We further investigated TCGA HNSCC (n = 566) using Xena to determine whether ORAI/STIM expression varies by anatomical subsite (Fig 1B). ORAI2, ORAI3, and STIM1 expression levels differed significantly across anatomic subdivisions (ORAI1: p > 0.05; ORAI2: p < 0.01; ORAI3: p < 0.01; STIM1: p < 0.05; STIM2: p > 0.05). Lip carcinomas expressed the highest levels of ORAI1, while tonsil and hypopharyngeal tumors expressed higher levels of ORAI2.

In the TCGA PanCancer Atlas HNSCC cohort (n = 523), genetic alterations in ORAI and STIM isoforms were relatively rare (Fig 1C). ORAI1 and ORAI3 were altered in <1% of cases, mostly through focal amplifications and missense mutations. ORAI2 was altered in ~3% of cases, predominantly through amplifications. Similarly, STIM1 was altered in <1% of cases, comprising deep deletions, truncating mutations, or missense variants. STIM2 showed alterations in ~2% of tumors, including amplifications, truncating variants, missense mutations, and structural rearrangements. These data suggest that SOCE dysregulation in HNSCC is not primarily driven by recurrent genomic alterations. In contrast, transcriptomic dysregulation was considerably more common (Fig 1D). mRNA expression relative to normal samples was altered in 7% of cases for ORAI1, 12% for ORAI2, 15% for ORAI3, and 6% for STIM2, primarily through upregulation. Notably, STIM1 was downregulated in 39% of tumors. These observations suggest that transcriptional dysregulation, rather than recurrent genomic alterations, is the dominant mechanism affecting SOCE components in HNSCC.

We further explored clinicopathological and molecular correlates of isoform expression. The expression of ORAI2 and STIM1 was strongly associated with advanced clinical stage (ORAI2: p < 0.05; STIM1: p < 0.01). While isoform expression remained relatively stable across stages I-III, we observed a significant increase in ORAI2 and a pronounced decrease in STIM1 in stage IVA/B tumors compared to stages I-III (Figure S1A in S2 File). STIM1 was significantly associated with pathological stage analysis (p < 0.05); STIM1 downregulation was most pronounced in stage IVB tumors, whereas ORAI isoforms were concomitantly elevated (Figure S1B in S2 File). Across tumor grade, ORAI1–3 expression correlated positively with dedifferentiation, which peaks in grade 4 tumors, whereas STIM1 showed an inverse association, with the lowest observed in grade 4 (ORAI1: p < 0.05; ORAI2: p < 0.001; ORAI3: p < 0.001; STIM1: p < 0.001) (Figure S2A in S2 File). Histological subtype analysis revealed that basaloid HNSCCs expressed significantly higher ORAI1–3 and lower STIM1 compared to conventional tumors, with STIM1 being the most discriminatory marker (*p* < 0.01) (Figure S2B in S2 File).

Additionally, we evaluated correlations with patient overall survival. High ORAI1 and STIM2 expression was significantly associated with improved overall survival. (ORAI1: p < 0.05; STIM2: p < 0.01). On the other hand, ORAI2, ORAI3, and STIM1 showed no significant association (Figure S3A-E in S2 File).

HPV-positive tumors displayed higher ORAI1, ORAI2, and STIM2 expression but reduced STIM1 expression (ORAI1: p < 0.01; ORAI2: p < 0.05; STIM1: p < 0.001; STIM2: p < 0.01) (Figure S4 in S2 File).

Finally, we examined the influence of key oncogenic drivers using PanCancer TCGA data via cBioPortal (n = 523). Tumors harboring PIK3CA mutations, which frequently occur at hotspot residues E542K, E545K (helical domain), and H1047R (kinase domain) and lead to constitutive PI3K/AKT pathway activation, exhibited significantly elevated STIM1 expression compared with wild-type cases (*p* < 0.001, *q* < 0.05). In contrast, ORAI1–3 mRNA levels were not significantly altered, although ORAI3 showed a trend toward upregulation (Figure S5A-E in S2 File). Stratification of STIM1 expression by PIK3CA mutation subtype revealed a striking enrichment in tumors harboring the H1047R hotspot mutation. Compared to wild-type tumors, H1047R cases demonstrated significantly elevated STIM1 expression (*p* < 0.05), whereas E542K and E545K mutants showed only modest, non-significant increases (Figure S5F in S2 File). These results suggest a mutation-specific effect of PIK3CA on STIM1 expression.

In contrast, EGFR expression correlated positively with STIM1 (Spearman r = 0.22, *p* < 0.001) and STIM2 (Spearman r = 0.14, *p* < 0.01), but negatively with ORAI1 (Spearman r = –0.26, *p* < 0.001) and ORAI3 (Spearman r = –0.18, *p* < 0.001), while no significant correlation was found with ORAI2 (Figure S6A-E in S2 File). These results indicate distinct oncogenic wiring of SOCE in HNSCC that reflects differential regulation by PIK3CA and EGFR.

In summary, our analysis of publicly available database datasets revealed a complex transcriptional reprogramming of SOCE in HNSCC, characterized by a dominant loss of STIM1 and a concurrent gain of ORAI isoforms. This signature is associated with aggressive disease and is modulated by key oncogenic pathways. This pattern suggests a potential adaptive mechanism in HNSCC progression, whereby tumors exploit SOCE components to fine-tune calcium signaling.

### Distinct SOCE profiles in HNSCC cell lines model molecular subtypes

To model the molecular heterogeneity of HNSCC in vitro, we selected FaDu and Detroit-562, two of the most widely used and well-characterized cell lines in HNSCC research. Both are HPV-negative and anatomically relevant, originating from the hypopharynx and pharynx, respectively, but differ markedly in disease context. FaDu is derived from a primary hypopharyngeal tumor, whereas Detroit-562 was established from a metastatic pleural effusion.

**Fig 2:**
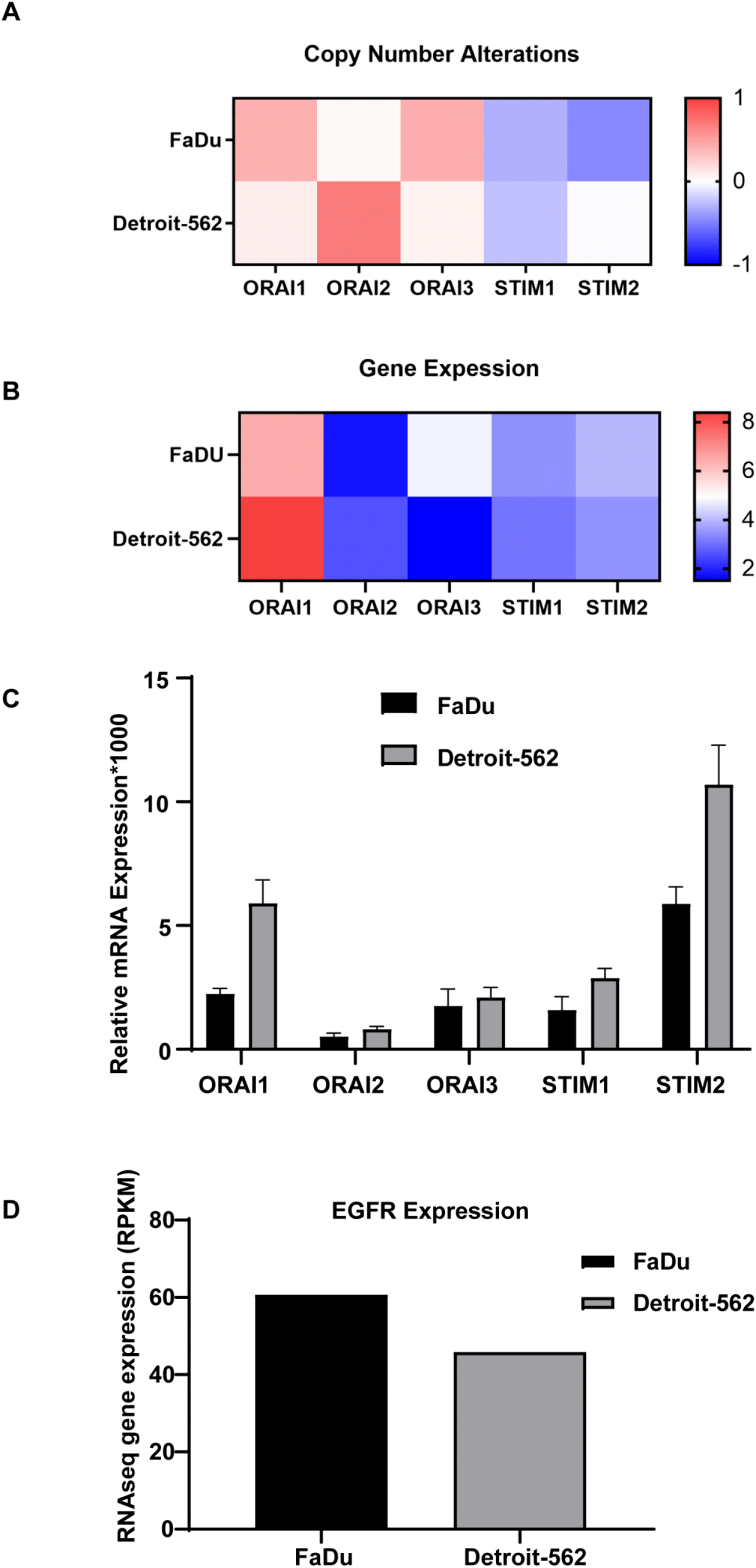
Variable molecular profiles of SOCE components and EGFR in HNSCC cell lines. **(A, B)** Copy number alterations (A) and mRNA expression levels (B) of ORAI1, ORAI2, ORAI3, STIM1, and STIM2 in FaDu and Detroit-562 HNSCC cell lines, retrieved from the Cancer Cell Line Encyclopedia (CCLE) using the UCSC Xena browser. Heatmaps illustrate relative gene copy number (A) and transcript abundance **(B)**. (C) Quantitative RT-PCR validation of ORAI and STIM isoform expression in FaDu and Detroit-562 cells (n = 3). Expression was normalized to GAPDH, and relative levels were calculated using the 2 ^(-ΔCt)^ method. Data represent mean ± SEM. **(D)** EGFR mRNA expression (RPKM) in FaDu and Detroit-562 cells, retrieved from CCLE/Xena. Values represent summary statistics; error bars are not shown.

The two models also represent distinct oncogenic backgrounds (Table 1). FaDu is characterized by higher EGFR expression and TP53 R248L and X225_splice mutations and demonstrates relative sensitivity to EGFR inhibition as reported in CCLE data and prior studies [33], which makes it a suitable EGFR-driven model. In contrast, Detroit-562 carries the canonical PIK3CA H1047R mutation with the TP53 R175H gain-of-function mutation, with lower EGFR expression, and serves as a representative PI3K-driven, metastatic subtype. EGFR expression from CCLE is presented as summary statistics in Fig 2D.

**Table 1:** Molecular characterization of HNSCC cell lines.

Cell line	EGFR expression	TP53 status	PIK3CA status
**FaDu**	High	R248L, X225 splice	WT
**Detroit-562**	Moderate	R175H (GOF)	H1047R (activating)

Summary of key molecular features of FaDu and Detroit-562 cells, including EGFR expression levels (RNA-seq, CCLE/Xena) and TP53 and PIK3CA mutation status (cBioPortal/CCLE). Abbreviations: WT, wild type. GOF = gain of function.

Copy number alterations and mRNA expression data for ORAI and STIM isoforms were obtained from the Cancer Cell Line Encyclopedia (CCLE) using the UCSC Xena Browser. Copy number alterations analysis showed differences between the two cell lines (Fig 2A). ORAI1 and ORAI3 copy numbers were higher in FaDu cells, while ORAI2 was higher in Detroit-562. In addition, STIM1 and STIM2 copy numbers were higher in Detroit-562 compared to FaDu. At the mRNA level, RNA-seq data indicated that ORAI1 and ORAI2 were more highly expressed in Detroit-562, whereas ORAI3, STIM1, and STIM2 were higher in FaDu (Fig 2B). However, quantitative real-time PCR (RT-qPCR) validation revealed higher expression of all ORAI and STIM isoforms in Detroit-562 cells compared to FaDu (Fig 2C).

Together, these findings characterize Detroit-562 as a “SOCE-high/PI3K-driven” model and FaDu as a “SOCE-low/EGFR-driven” model. Collectively, they provide complementary experimental platforms that represent primary vs metastatic contexts, EGFR- vs PI3K-driven oncogenic backgrounds, and SOCE-low vs SOCE-high expression states of HNSCC, which offer a robust system to probe functional SOCE dependencies.

### Pharmacological inhibition of SOCE using 2-APB attenuates HNSCC cell viability

To assess the effect of SOCE inhibition on HNSCC cell proliferation, 2-aminoethoxydiphenyl borate (2-APB), a widely used small-molecule SOCE inhibitor, was used. 2-APB interferes with ORAI channel activity and disrupts STIM–ORAI coupling, thereby attenuating calcium influx [30,31]. Although 2-APB may have additional channel targets, it remains a well-characterized and accepted tool for probing SOCE dependency in cancer models.

**Fig 3:**
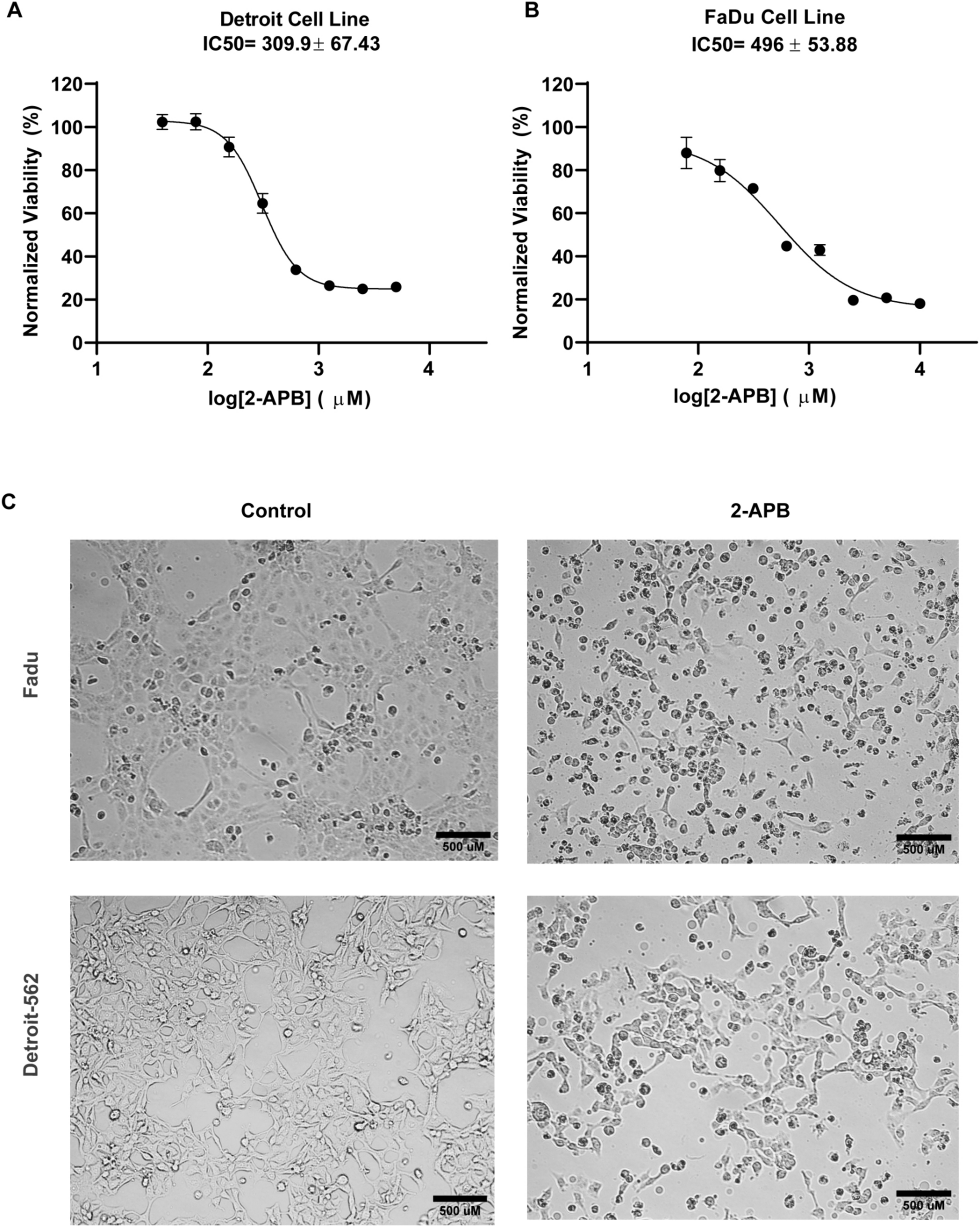
Pharmacological inhibition of SOCE with 2-APB induces cytotoxic effects in HNSCC cells. **(A, B)** Dose–response curves of FaDu (A) and Detroit-562 (B) cell lines treated with increasing concentrations of 2-APB for 48 hours. Cell viability was assessed using the MTT assay. Viability was normalized to vehicle-treated control, and IC₅₀ values were calculated using non-linear regression (log[2-APB] vs. % viability) in GraphPad Prism 8.0. Data represent the mean ± SD of at least three independent experiments performed in sextuplicate. **(C)** Representative bright-field images of FaDu and Detroit-562 cells after 24-hour treatment with 2-APB at corresponding IC₅₀ concentrations. Representative images are shown to illustrate qualitative morphological changes (cell shrinkage, rounding, detachment) associated with cytotoxicity. Quantitative assessment of viability at 48 hours is provided in panels A-B. Images were captured at 10X magnification; scale bar = 500 µm.

FaDu and Detroit-562 cells were treated with increasing concentrations of 2-APB for 48 hours, followed by MTT assay to assess viability. The 48-hour time point was chosen for IC₅₀ determination, as it captures the cumulative loss of metabolic activity required for reliable quantification of drug response. Dose–response curves demonstrated a concentration-dependent reduction in cell viability in both cell lines (Fig 3A and 3B). Cell viability was normalized to vehicle-treated control, and IC₅₀ values were calculated using model fitting based on log-transformed 2-APB concentrations. The IC₅₀ for Detroit-562 cells was 309.9 µM, compared to 496.7 µM for FaDu cells, which indicates higher sensitivity of Detroit-562 cells to 2-APB treatment.

To display the earliest morphological signs of cytotoxicity, which typically precede measurable reductions in viability**,** bright-field images were captured 24 hours after treatment at IC₅₀ concentrations for each cell line. Both FaDu and Detroit-562 cells showed signs of cytotoxicity, which includes cell shrinkage, rounding, loss of attachment, and the presence of cellular debris (Fig 3C).

These results indicate that pharmacological inhibition of SOCE with 2-APB suppresses HNSCC cell viability in a dose-dependent manner, with greater sensitivity observed in the SOCE-high Detroit-562 model.

### Pharmacological inhibition of SOCE using 2-APB inhibits migration of HNSCC cells

SOCE has been shown to have an important role in potentiating cell motility and metastasis. To evaluate the effect of ORAI inhibition on cell migration, a wound healing assay was utilized in FaDu and Detroit-562 cells. To ensure our assay specifically measured migration and was not confounded by effects on cell viability, we first optimized the experimental timeline. We evaluated time points between 6 and 24 hours and selected a 12-hour endpoint, as this window was sufficient to observe migration in controls while avoiding the pronounced cytotoxicity and monolayer disintegration observed at 24 hours.

Confluent monolayers were manually scratched and immediately treated with 2-APB. We utilized lower, sub-cytotoxic concentrations (200 µM for FaDu and 100 µM for Detroit-562) to preserve monolayer integrity and reliably quantify motility, as IC₅₀ concentrations induced substantial cell death.

**Fig 4:**
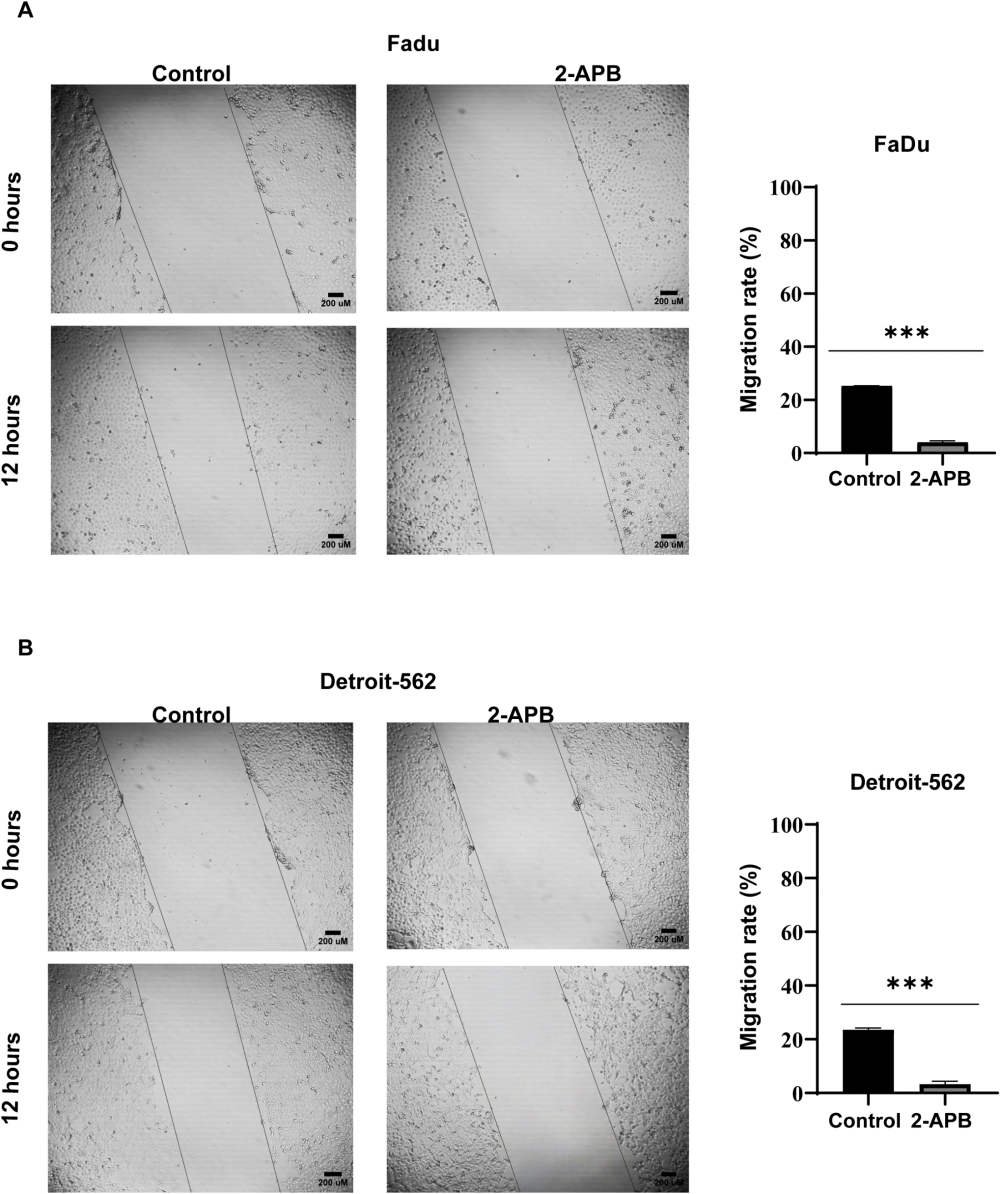
Pharmacological inhibition of SOCE with 2-APB suppresses migration of HNSCC cells. **(A, B)** The effect of 2-APB on cell migration was assessed using a wound healing assay in FaDu (A) and Detroit-562 (B) cells. Cells were treated with 200 µM (FaDu) or 100 µM (Detroit-562) 2-APB for 12 hours. Representative bright-field images were captured at 0 and 12 hours using a light microscope at 5 × magnification. Scale bar = 200 µm. Migration rates (%) were calculated using ImageJ software with the formula: Migration (%) = [(Distance at 0 h – Distance at 12 **h)** / Distance at 0 **h]** × 100. Data represent the mean ± SEM of at least three independent experiments performed in triplicate. Statistical significance was assessed using Student’s t-test. ***p < 0.001.

Representative bright-field images taken at 0 and 12-hours post-treatment showed the effect of 2-APB on wound closure. By 12 hours, untreated control cells had migrated into the scratch region. In contrast, 2-APB-treated cells showed markedly reduced wound closure (Fig 4A and 4B).

Quantitative analysis showed reduced migration in 2-APB-treated cells. In FaDu cells, the migration rate decreased from 25.24% (control) to 4.00% (2-APB-treated). Similarly, Detroit-562 cells showed a reduction from 23.58% (control) to 3.28% (2-APB-treated) (Fig 4A and 4B).

These results indicate that pharmacological SOCE inhibition impairs HNSCC cell migration under non-cytotoxic conditions, which reinforces the role of SOCE in motility.

### Pharmacological inhibition of SOCE using 2-APB inhibits clonogenic potential of HNSCC cells

To assess the effect of 2-APB on HNSCC survival, proliferative potential, and recovery, colony formation assays were employed in FaDu and Detroit-562 cell lines using two experimental settings: long-term exposure and short-term treatment followed by recovery.

**Fig 5:**
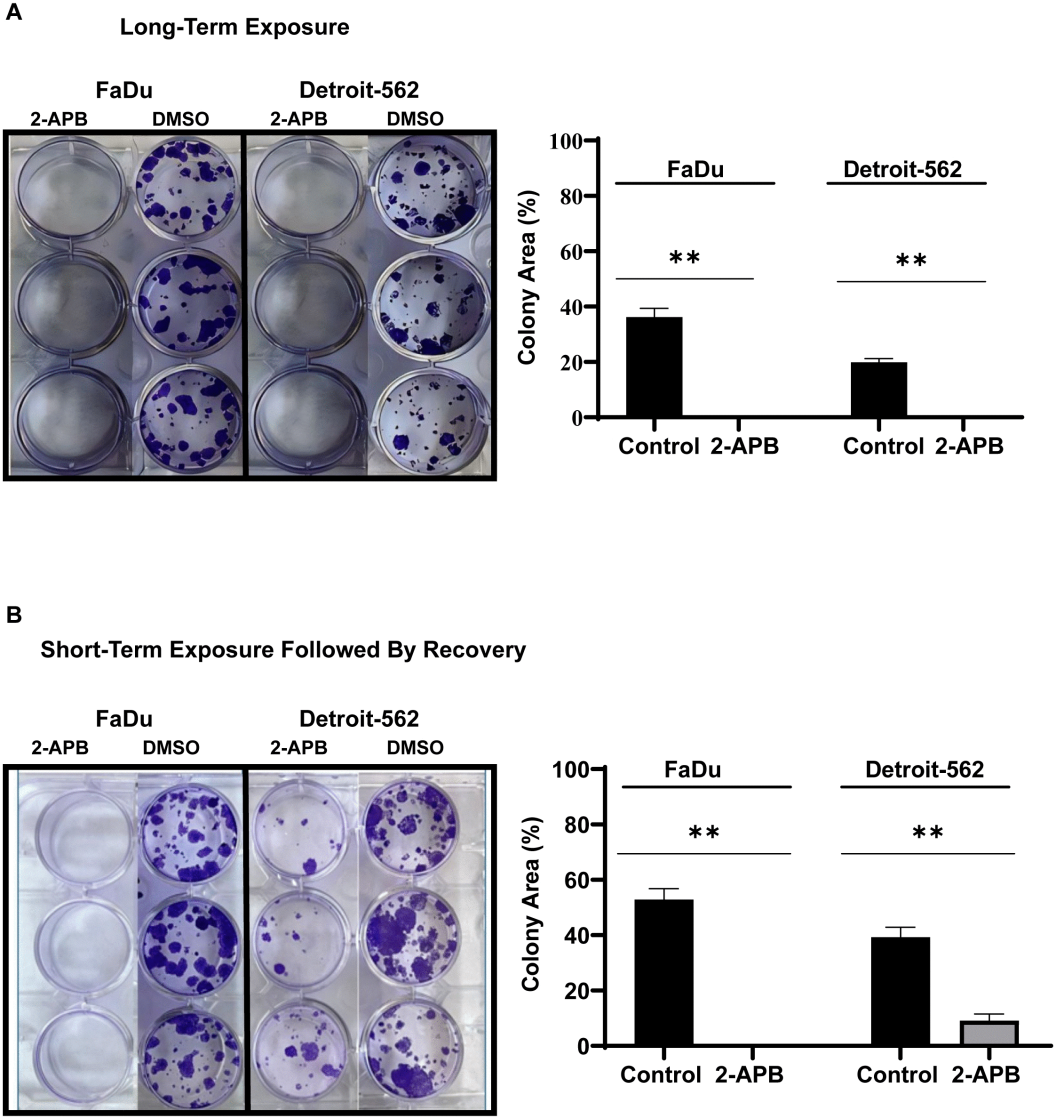
2-APB impairs clonogenic potential of HNSCC cells under long- and short-term exposure conditions. **(A)** FaDu and Detroit-562 cells were exposed to continuous 2-APB treatment (200 µM and 100 µM, respectively) for 3–4 weeks to assess long-term clonogenic survival. Representative images show the absence of colony formation in treated cells compared to robust colony growth in untreated controls. **(B)** Cells were treated with 2-APB for 72 hours, after which the drug was removed and replaced with drug-free medium. Following a 3–4 weeks recovery period, colonies were fixed and stained. Colony area (%) was quantified using ImageJ, and results represent the mean ± SEM of at least three independent experiments. Statistical significance was assessed using a Student’s t-test. **p < 0.01.

For the long-term exposure experiments, cells were seeded at low density (250 cells/well) in 12-well plates and treated with 200 µM 2-APB for FaDu and 100 µM for Detroit-562. After 3–4 weeks, colonies were fixed and stained. In both cell lines, 2-APB-treated cells failed to form visible colonies, whereas colonies were observed in untreated controls (Fig 5A). Quantitative analysis showed that the control groups covered a significantly larger surface area (FaDu: 33.991%; Detroit-562: 33.082%), while treated wells showed no detectable cell growth.

For the short-term exposure experiments, FaDu and Detroit-562 cells were treated with 2-APB at the same concentrations for 72 hours, after which the media was replaced with drug-free medium for 3–4 weeks to assess their ability to recover and form colonies. FaDu cells failed to form colonies following short-term treatment, which indicates a loss of clonogenic potential. In contrast, Detroit-562 cells showed partial colony formation after drug removal (Fig 5B). Quantitative analysis showed a significant reduction in colony area in both cell lines. Control FaDu cells covered 52.878% of the well area, but no growth was observed in the treated group. In Detroit-562 cells, the control group covered 39.263%, whereas the treated group formed fewer and smaller colonies, covering 9.135% of the well area.

Together, these results suggest that SOCE inhibition with 2-APB reduces the colony-forming ability in HNSCC cells. Notably, FaDu cells exhibited a complete and irreversible loss of clonogenic potential, whereas Detroit-562 cells retained limited recovery capacity, which suggests subtype-specific differences in dependence on SOCE for long-term survival.

## Discussion

Store-operated calcium entry (SOCE) has emerged as a critical regulator of cancer progression [25,34], yet its role in head and neck squamous cell carcinoma (HNSCC) has remained poorly defined. Here, we present an isoform-level characterization of SOCE remodeling in HNSCC and demonstrate context-dependent therapeutic vulnerabilities that challenge the traditional view of uniform SOCE hyperactivation in cancer.

Our TCGA analysis identified a complex pattern of SOCE remodeling in HNSCC. While ORAI1–3 and STIM2 are broadly upregulated, STIM1, the canonical SOCE activator, is downregulated in nearly 40% of tumors, particularly in advanced and poorly differentiated tumors. This suggests an adaptive “isoform switch” that may shift from STIM1-dependent, high-threshold calcium entry to STIM2-mediated basal signaling. Unlike STIM1, which activates only after substantial ER Ca^2+^ depletion to drive proliferative bursts, STIM2 senses smaller fluctuations and remains partially active at baseline, which sustains low-level Ca^2+^ influx that promotes stress adaptation and metabolic flexibility while minimizing cytotoxic calcium overload [15,16,35,36]. The concurrent upregulation of ORAI2 and ORAI3, which form constitutively active, oxidation-resistant heteromers [23,24,37–39], further supports this metabolic adaptation. Evidence from other cancers supports this paradigm. In hepatocellular carcinoma, STIM1 acts as a metabolic checkpoint that supports proliferation and anoikis resistance during early tumorigenesis but becomes attenuated during epithelial–mesenchymal transition (EMT) to facilitate metabolic reprogramming that favors invasion and metastasis [40]. In colorectal cancer, STIM2 loss enhances growth and metastatic progression through metabolic reprogramming and ER stress adaptation [41]. By analogy, HNSCC progression appears to use a similar isoform switch. STIM1 downregulation coupled with STIM2/ORAI1–3 upregulation reprograms calcium signaling from a proliferative state toward one that sustains stress adaptation and invasive behavior.

A key observation from our TCGA analysis is that dominant oncogenic drivers are associated with distinct patterns of SOCE remodeling. PIK3CA-mutant tumors, particularly those with H1047R, showed elevated STIM1. This observation aligns with the established oncogenic functions of PIK3CA mutations in HNSCC. The first functional characterization demonstrated that mutant PIK3CA confers growth factor independence, enhanced migration, and invasion through constitutive PI3K/AKT activation [7], and subsequent studies established PIK3CA as a key driver of oral cancer through sustained activation of survival and metabolic pathways [8]. This oncogenic signaling is critically regulated upstream by RAS, a central driver of HNSCC tumorigenesis that integrates growth factor signals to activate the PI3K pathway [9]. The interplay between RAS and PI3K signaling, subject to regulatory control by microRNAs [42], creates a complex oncogenic network. The coordinated activation of this signaling network may create a metabolic demand with increased SOCE dependence that requires enhanced calcium influx for sustained growth and survival [43,44], a state we may call “SOCE addiction.” By contrast, EGFR expression correlated positively with STIM1 but negatively with ORAI1/3, producing a “STIM1-high/ORAI-low” signature. This pattern aligns with the complex, differential regulation of EGFR signaling in HNSCC [6]. We propose that in this context, STIM1 is co-opted into non-canonical functions at ER–plasma membrane junctions to potentiate growth factor signaling [36,45], while ORAI suppression prevents calcium-induced apoptosis [46–48]. Together, our findings reveal that SOCE remodeling in HNSCC is not uniform but instead reflects both disease stage and oncogenic context, which appears to generate distinct vulnerabilities that may guide biomarker-informed therapy.

Beyond direct mutational activation, the oncogenic landscape of HNSCC is further shaped by post-transcriptional regulatory mechanisms. MicroRNAs have emerged as critical modulators of key oncogenic pathways in oral and head and neck cancers, including regulation of RAS family oncogenes [42] and epithelial-mesenchymal plasticity through the miR-200 family [49]. Whether such regulatory layers influence the transcriptional remodeling of SOCE components observed in advanced HNSCC, particularly the coordinated downregulation of STIM1 and upregulation of ORAI isoforms, remains an open question that warrants further investigation.

FaDu and Detroit-562 cell lines were selected to model the molecular diversity identified in our TCGA analysis. FaDu (primary origin, EGFR-high, PIK3CA-wild-type) represents the “SOCE-low/EGFR-driven” subtype, while Detroit-562 (metastatic origin, PIK3CA H1047R mutant) represents the “SOCE-high/PI3K-addicted” phenotype. This selected pairing provides complementary experimental platforms that reflect both the genomic landscape and disease progression states of HNSCC. RT-qPCR validation confirmed that Detroit-562 cells express higher levels of all SOCE isoforms compared to FaDu, consistent with their PIK3CA-mutant status and metastatic origin. This molecular distinction was associated with functional dependencies. Detroit-562 demonstrated greater sensitivity to pharmacological SOCE inhibition using 2-APB (IC₅₀ 310 µM vs. 497 µM for FaDu), which is consistent with our hypothesis that SOCE-high tumors may represent a tractable therapeutic target. SOCE inhibition nearly abolished wound closure in both models, which indicates that calcium influx contributes to migratory capacity. Directed cell migration is a calcium-regulated process requiring coordinated cytoskeletal dynamics, including actin polymerization, actomyosin contractility, and adhesion turnover [50]. SOCE, mediated by ORAI and STIM proteins, is well-established as a regulator of these processes [34]. The marked reduction in migration observed following 2-APB treatment is therefore consistent with impaired calcium-dependent cytoskeletal processes. The morphological changes observed, such as cell rounding, membrane retraction, and detachment, are likewise consistent with altered cytoskeletal organization downstream of SOCE signaling [34,51,52]. This effect was observed across EGFR- and PI3K-driven contexts, which underscores SOCE’s conserved role in metastatic dissemination. While migration is a necessary component of the invasion-metastasis cascade, future studies using Transwell invasion assays, 3D spheroid invasion models, and in vivo metastasis assays will be needed to definitively establish whether SOCE inhibition suppresses invasive and metastatic behavior in HNSCC. Nevertheless, the observation that SOCE blockade disrupts migration, a prerequisite for metastatic dissemination, combined with TCGA evidence of SOCE upregulation in metastatic HNSCC tissues, supports a potential role for SOCE in metastatic progression that warrants further investigation. Clonogenic assays added another layer of insight. Continuous SOCE inhibition abolished colony formation in both cell lines, which supports that long-term proliferative survival is SOCE dependent**.** Notably, Detroit-562 cells demonstrated partial clonogenic recovery after short-term 2-APB exposure, despite showing greater acute sensitivity in viability assays. This apparent paradox resolves when recognizing that these assays measure distinct biological properties. Acute viability assays capture immediate SOCE dependence for baseline survival, whereas recovery assays assess resilience to transient stress and the capacity to resume proliferation after drug withdrawal. Detroit-562's PIK3CA-mutant, metastatic context likely enables compensatory survival signaling during transient SOCE blockade, particularly through constitutive PI3K/AKT/mTOR pathway activation, which allows clonogenic recovery once the inhibitor is removed [27,43,44]. In contrast, FaDu's EGFR-driven signaling may require continuous SOCE-EGFR coordination for proliferative capacity, such that transient disruption causes irreversible decoupling and loss of clonogenic potential [53,54]. These findings suggest that SOCE dependencies are context-specific: while SOCE-high tumors show greater acute vulnerability, they may also possess adaptive mechanisms conferring resilience to transient inhibition. Therapeutic strategies may therefore need to account for both acute sensitivity and adaptive resilience, potentially requiring sustained SOCE blockade or combination with PI3K/AKT pathway inhibitors to prevent recovery in PIK3CA-mutant tumors.

Together, our findings propose a model in which SOCE remodeling reflects oncogenic context, which suggests that PIK3CA-mutant tumors are predisposed to SOCE dependence, while EGFR-driven cancers may engage alternative signaling. This model provides a rationale for future investigation into biomarker-guided patient stratification. For instance, the STIM1-low/ORAI-high signature, enriched in advanced disease, may identify patients who benefit from SOCE-targeted therapies. Furthermore, the functional SOCE dependence observed in PIK3CA-mutant models warrants testing; dual blockade of SOCE and PI3K/AKT may be required to prevent adaptive resistance and achieve durable responses, while the distinct pattern in EGFR-driven contexts suggests SOCE inhibition could be explored in combination with EGFR-targeted agents. Finally, the observed prognostic association of high ORAI1 and STIM2 with improved survival likely reflects SOCE’s essential role in T cell activation and antitumor immunity [25,55], which raises the possibility that SOCE modulation could synergize with immunotherapy. As SOCE dysregulation occurs mainly at the transcriptional level, it represents a dynamic and potentially reversible vulnerability for biomarker-driven therapeutic strategies.

While our study identifies associations that support a context-dependent role for SOCE in HNSCC, several limitations should be considered when interpreting our findings. 2-APB, although well established as a SOCE inhibitor, is not isoform-specific and may exert off-target effects. While we did not perform calcium imaging to directly measure store-operated Ca^2+^ influx, our comprehensive functional validation, spanning acute (migration) and chronic (clonogenicity) endpoints across two cell lines with differential SOCE expression, provides strong evidence of effective SOCE pathway inhibition. The correlation between baseline SOCE mRNA levels and 2-APB sensitivity supports on-target inhibition, though future studies employing ratiometric Ca^2+^ imaging (e.g., Fura-2 measurements) would provide direct biochemical confirmation of channel blockade. While our consistent phenotypes across viability, migration, and clonogenic assays support SOCE dependence, definitive attribution to individual ORAI or STIM isoforms will require isoform-specific inhibitors or genetic perturbation strategies. Functional assays were limited to two cell lines. Although these were selected to represent distinct molecular contexts, they cannot capture the full heterogeneity of HNSCC, and in vivo validation is needed to assess therapeutic efficacy without compromising host immunity. Furthermore, while our study relies on mRNA expression as the primary molecular readout, the established correlation between ORAI/STIM mRNA and protein levels in cancer contexts [25,26], combined with our functional validation across multiple phenotypic assays, provides robust support for our conclusions. Future studies incorporating protein-level quantification via Western blot or immunohistochemistry in patient tissues would further validate the isoform-specific remodeling patterns identified here. Our in vitro functional analysis was focused on 2D models of viability, migration, and clonogenic survival. While informative, these assays do not directly model critical processes such as proteolytic invasion through basement membranes or the complex steps of metastasis in vivo. Finally, although our functional assays indicate SOCE-dependent cytoskeletal impairment, we did not directly visualize F-actin architecture (e.g., phalloidin staining). Such structural analyses would provide complementary mechanistic insight and represent a logical direction for future studies to delineate the specific cytoskeletal pathways regulated by SOCE in HNSCC. Despite these limitations, our integrated approach provides a strong foundation for these future investigations.

## Conclusion

Through integrated genomic analysis and functional validation, our study suggests that SOCE in HNSCC may represent a context-dependent vulnerability rather than a uniformly activated pathway. We observed that tumors undergo isoform-specific remodeling, with STIM1 downregulation and ORAI2/3 upregulation, which may reflect an adaptive switch that favors survival and invasion in advanced disease. Importantly, our TCGA analyses and cell line studies provide evidence that supports a model in which PIK3CA-mutant tumors are predisposed to SOCE dependence, while EGFR-driven cancers appear to engage alternative calcium coupling strategies. Functionally, SOCE inhibition suppressed viability, migration, and clonogenic growth, suggesting calcium signaling as a tractable therapeutic axis. These findings provide a rationale for biomarker-guided patient stratification and suggest that SOCE blockade, potentially in combination with PI3K/AKT or EGFR-targeted therapies, could be explored as precision strategies for aggressive HNSCC.

## Supporting information

S1 File Supplementary Tables S1–S2. (DOCX)

S2 File Supplementary Figures S1–S7. (DOCX)

## References

[1] Johnson DE, Burtness B, Leemans CR, Lui VWY, Bauman JE, Grandis JR. Head and neck squamous cell carcinoma. Nat Rev Dis Primers. 2020;6(1):92. doi: 10.1038/s41572-020-00224-3 33243986 PMC7944998

[2] Sung H, Ferlay J, Siegel RL, Laversanne M, Soerjomataram I, Jemal A, et al. Global Cancer Statistics 2020: GLOBOCAN Estimates of Incidence and Mortality Worldwide for 36 Cancers in 185 Countries. CA Cancer J Clin. 2021;71(3):209–49. doi: 10.3322/caac.21660 33538338

[3] Economopoulou P, de Bree R, Kotsantis I, Psyrri A. Diagnostic Tumor Markers in Head and Neck Squamous Cell Carcinoma (HNSCC) in the Clinical Setting. Front Oncol. 2019;9:827. doi: 10.3389/fonc.2019.00827 31555588 PMC6727245

[4] Baniebrahimi G, Mir F, Khanmohammadi R. Cancer stem cells and oral cancer: insights into molecular mechanisms and therapeutic approaches. Cancer Cell Int. 2020;20:113. doi: 10.1186/s12935-020-01192-0 32280305 PMC7137421

[5] Leemans CR, Snijders PJF, Brakenhoff RH. The molecular landscape of head and neck cancer. Nat Rev Cancer. 2018;18(5):269–82. doi: 10.1038/nrc.2018.11 29497144

[6] Chakkarappan SR, Umadharshini KV, Dhamodharan S, Rose MM, Gopu G, Murugan AK, et al. Super enhancer loci of EGFR regulate EGFR variant 8 through enhancer RNA and strongly associate with survival in HNSCCs. Mol Genet Genomics. 2024;299(1):3. doi: 10.1007/s00438-023-02089-z 38236481

[7] Murugan AK, Hong NT, Fukui Y, Munirajan AK, Tsuchida N. Oncogenic mutations of the PIK3CA gene in head and neck squamous cell carcinomas. Int J Oncol. 2008;32(1):101–11. 18097548

[8] Murugan AK, Munirajan AK, Tsuchida N. Genetic deregulation of the PIK3CA oncogene in oral cancer. Cancer Lett. 2013;338(2):193–203. doi: 10.1016/j.canlet.2013.04.005 23597702

[9] Murugan AK, Munirajan AK, Tsuchida N. Ras oncogenes in oral cancer: the past 20 years. Oral Oncol. 2012;48(5):383–92. doi: 10.1016/j.oraloncology.2011.12.006 22240207

[10] Zheng S, Wang X, Zhao D, Liu H, Hu Y. Calcium homeostasis and cancer: insights from endoplasmic reticulum-centered organelle communications. Trends Cell Biol. 2023;33(4):312–23. doi: 10.1016/j.tcb.2022.07.004 35915027

[11] Prakriya M, Lewis RS. Store-Operated Calcium Channels. Physiol Rev. 2015;95(4):1383–436. doi: 10.1152/physrev.00020.2014 26400989 PMC4600950

[12] Soboloff J, Rothberg BS, Madesh M, Gill DL. STIM proteins: dynamic calcium signal transducers. Nat Rev Mol Cell Biol. 2012;13(9):549–65. doi: 10.1038/nrm3414 22914293 PMC3458427

[13] Trebak M, Putney JW Jr. ORAI Calcium Channels. Physiology (Bethesda). 2017;32(4):332–42. 28615316 10.1152/physiol.00011.2017 PMC5545608

[14] Hou X, Pedi L, Diver MM, Long SB. Crystal structure of the calcium release-activated calcium channel Orai. Science. 2012;338(6112):1308–13. doi: 10.1126/science.1228757 23180775 PMC3695727

[15] Brandman O, Liou J, Park WS, Meyer T. STIM2 is a feedback regulator that stabilizes basal cytosolic and endoplasmic reticulum Ca2+ levels. Cell. 2007;131(7):1327–39. doi: 10.1016/j.cell.2007.11.039 18160041 PMC2680164

[16] Ahmad M, Ong HL, Saadi H, Son G-Y, Shokatian Z, Terry LE, et al. Functional communication between IP3R and STIM2 at subthreshold stimuli is a critical checkpoint for initiation of SOCE. Proc Natl Acad Sci U S A. 2022;119(3):e2114928118. doi: 10.1073/pnas.2114928118 35022238 PMC8784118

[17] Spassova MA, Soboloff J, He L-P, Xu W, Dziadek MA, Gill DL. STIM1 has a plasma membrane role in the activation of store-operated Ca(2+) channels. Proc Natl Acad Sci U S A. 2006;103(11):4040–5. doi: 10.1073/pnas.0510050103 16537481 PMC1449642

[18] Roos J, DiGregorio PJ, Yeromin AV, Ohlsen K, Lioudyno M, Zhang S, et al. STIM1, an essential and conserved component of store-operated Ca2+ channel function. J Cell Biol. 2005;169(3):435–45. doi: 10.1083/jcb.200502019 15866891 PMC2171946

[19] Liou J, Kim ML, Heo WD, Jones JT, Myers JW, Ferrell JE Jr, et al. STIM is a Ca2+ sensor essential for Ca2+-store-depletion-triggered Ca2+ influx. Curr Biol. 2005;15(13):1235–41. doi: 10.1016/j.cub.2005.05.055 16005298 PMC3186072

[20] Mercer JC, Dehaven WI, Smyth JT, Wedel B, Boyles RR, Bird GS, et al. Large store-operated calcium selective currents due to co-expression of Orai1 or Orai2 with the intracellular calcium sensor, Stim1. J Biol Chem. 2006;281(34):24979–90. doi: 10.1074/jbc.M604589200 16807233 PMC1633822

[21] Soboloff J, Spassova MA, Tang XD, Hewavitharana T, Xu W, Gill DL. Orai1 and STIM reconstitute store-operated calcium channel function. J Biol Chem. 2006;281(30):20661–5. doi: 10.1074/jbc.C600126200 16766533

[22] Zhang X, Xin P, Yoast RE, Emrich SM, Johnson MT, Pathak T, et al. Distinct pharmacological profiles of ORAI1, ORAI2, and ORAI3 channels. Cell Calcium. 2020;91:102281. doi: 10.1016/j.ceca.2020.102281 32896813 PMC7654283

[23] Yoast RE, Emrich SM, Trebak M. The anatomy of native CRAC channel(s). Curr Opin Physiol. 2020;17:89–95. doi: 10.1016/j.cophys.2020.07.012 32999945 PMC7521663

[24] Bogeski I, Kummerow C, Al-Ansary D, Schwarz EC, Koehler R, Kozai D, et al. Differential redox regulation of ORAI ion channels: a mechanism to tune cellular calcium signaling. Sci Signal. 2010;3(115):ra24. doi: 10.1126/scisignal.2000672 20354224

[25] Chen Y-F, Lin P-C, Yeh Y-M, Chen L-H, Shen M-R. Store-Operated Ca2+ Entry in Tumor Progression: From Molecular Mechanisms to Clinical Implications. Cancers (Basel). 2019;11(7):899. doi: 10.3390/cancers11070899 31252656 PMC6678533

[26] Wang Y-Y, Wang W-C, Su C-W, Hsu C-W, Yuan S-S, Chen Y-K. Expression of Orai1 and STIM1 in human oral squamous cell carcinogenesis. J Dent Sci. 2022;17(1):78–88. doi: 10.1016/j.jds.2021.07.004 35028023 PMC8739746

[27] Singh AK, Roy NK, Bordoloi D, Padmavathi G, Banik K, Khwairakpam AD, et al. Orai-1 and Orai-2 regulate oral cancer cell migration and colonisation by suppressing Akt/mTOR/NF-κB signalling. Life Sci. 2020;261:118372. doi: 10.1016/j.lfs.2020.118372 32882268

[28] Hegde M, Daimary UD, Jose S, Sajeev A, Chinnathambi A, Alharbi SA, et al. Differential Expression of Genes Regulating Store-operated Calcium Entry in Conjunction With Mitochondrial Dynamics as Potential Biomarkers for Cancer: A Single-Cell RNA Analysis. Front Genet. 2022;13:866473. doi: 10.3389/fgene.2022.866473 35711942 PMC9197647

[29] Lee SH, Rigas NK, Lee C-R, Bang A, Srikanth S, Gwack Y, et al. Orai1 promotes tumor progression by enhancing cancer stemness via NFAT signaling in oral/oropharyngeal squamous cell carcinoma. Oncotarget. 2016;7(28):43239–55. doi: 10.18632/oncotarget.9755 27259269 PMC5190020

[30] DeHaven WI, Smyth JT, Boyles RR, Bird GS, Putney JW Jr. Complex actions of 2-aminoethyldiphenyl borate on store-operated calcium entry. J Biol Chem. 2008;283(28):19265–73. doi: 10.1074/jbc.M801535200 18487204 PMC2443677

[31] Wang Y, Deng X, Zhou Y, Hendron E, Mancarella S, Ritchie MF, et al. STIM protein coupling in the activation of Orai channels. Proc Natl Acad Sci U S A. 2009;106(18):7391–6. doi: 10.1073/pnas.0900293106 19376967 PMC2678612

[32] Franken NAP, Rodermond HM, Stap J, Haveman J, van Bree C. Clonogenic assay of cells in vitro. Nat Protoc. 2006;1(5):2315–9. doi: 10.1038/nprot.2006.339 17406473

[33] Sun C, Han C, Jiang Y, Han N, Zhang M, Li G, et al. Inhibition of GRP78 abrogates radioresistance in oropharyngeal carcinoma cells after EGFR inhibition by cetuximab. PLoS One. 2017;12(12):e0188932. doi: 10.1371/journal.pone.0188932 29232380 PMC5726659

[34] Hammad AS, Machaca K. Store Operated Calcium Entry in Cell Migration and Cancer Metastasis. Cells. 2021;10(5):1246. doi: 10.3390/cells10051246 34069353 PMC8158756

[35] Subedi KP, Ong HL, Son G-Y, Liu X, Ambudkar IS. STIM2 Induces Activated Conformation of STIM1 to Control Orai1 Function in ER-PM Junctions. Cell Rep. 2018;23(2):522–34. doi: 10.1016/j.celrep.2018.03.065 29642009

[36] Ahmad M, Narayanasamy S, Ong HL, Ambudkar I. STIM proteins and regulation of SOCE in ER-PM junctions. Biomolecules. 2022;12(8). 10.3390/biom12081152 PMC9405863 36009047

[37] Yoast RE, Emrich SM, Zhang X, Xin P, Johnson MT, Fike AJ, et al. The native ORAI channel trio underlies the diversity of Ca2+ signaling events. Nat Commun. 2020;11(1):2444. doi: 10.1038/s41467-020-16232-6 32415068 PMC7229178

[38] Alansary D, Bogeski I, Niemeyer BA. Facilitation of Orai3 targeting and store-operated function by Orai1. Biochim Biophys Acta. 2015;1853(7):1541–50. doi: 10.1016/j.bbamcr.2015.03.007 25791427

[39] Kouba S, Buscaglia P, Guéguinou M, Ibrahim S, Félix R, Guibon R, et al. Pivotal role of the ORAI3-STIM2 complex in the control of mitotic death and prostate cancer cell cycle progression. Cell Calcium. 2023;115:102794. doi: 10.1016/j.ceca.2023.102794 37597301

[40] Zhao H, Yan G, Zheng L, Zhou Y, Sheng H, Wu L, et al. STIM1 is a metabolic checkpoint regulating the invasion and metastasis of hepatocellular carcinoma. Theranostics. 2020;10(14):6483–99. doi: 10.7150/thno.44025 32483465 PMC7255033

[41] Pathak T, Benson JC, Johnson MT, Xin P, Abdelnaby AE, Walter V, et al. Loss of STIM2, but not of STIM1, drives colorectal cancer metastasis through metabolic reprogramming and the ATF4 ER stress pathway. Sci Signal. 2025;18(892):eads6550. doi: 10.1126/scisignal.ads6550 40554601 PMC12507093

[42] Murugan AK, Munirajan AK, Alzahrani AS. MicroRNAs: Modulators of the Ras Oncogenes in Oral Cancer. J Cell Physiol. 2016;231(7):1424–31. doi: 10.1002/jcp.25269 26620726

[43] Hoxhaj G, Manning BD. The PI3K-AKT network at the interface of oncogenic signalling and cancer metabolism. Nat Rev Cancer. 2020;20(2):74–88. doi: 10.1038/s41568-019-0216-7 31686003 PMC7314312

[44] Divolis G, Mavroeidi P, Mavrofrydi O, Papazafiri P. Differential effects of calcium on PI3K-Akt and HIF-1α survival pathways. Cell Biol Toxicol. 2016;32(5):437–49. doi: 10.1007/s10565-016-9345-x 27344565

[45] Bai W, Yan C, Yang Y, Sang L, Hao Q, Yao X, et al. EGF/EGFR-YAP1/TEAD2 signaling upregulates STIM1 in vemurafenib resistant melanoma cells. FEBS J. 2024;291(22):4969–83. doi: 10.1111/febs.17272 39298503

[46] Tanwar J, Motiani RK. Role of SOCE architects STIM and Orai proteins in Cell Death. Cell Calcium. 2018;69:19–27. doi: 10.1016/j.ceca.2017.06.002 28629579

[47] Flourakis M, Lehen’kyi V, Beck B, Raphaël M, Vandenberghe M, Abeele FV, et al. Orai1 contributes to the establishment of an apoptosis-resistant phenotype in prostate cancer cells. Cell Death Dis. 2010;1(9):e75. doi: 10.1038/cddis.2010.52 21364678 PMC3032347

[48] Benson JC, Trebak M. Too much of a good thing: The case of SOCE in cellular apoptosis. Cell Calcium. 2023;111:102716. doi: 10.1016/j.ceca.2023.102716 36931194 PMC10481469

[49] Arunkumar G, Deva Magendhra Rao AK, Manikandan M, Prasanna Srinivasa Rao H, Subbiah S, Ilangovan R, et al. Dysregulation of miR-200 family microRNAs and epithelial-mesenchymal transition markers in oral squamous cell carcinoma. Oncol Lett. 2018;15(1):649–57. doi: 10.3892/ol.2017.7296 29375721 PMC5766066

[50] Tsai F-C, Kuo G-H, Chang S-W, Tsai P-J. Ca2+ signaling in cytoskeletal reorganization, cell migration, and cancer metastasis. Biomed Res Int. 2015;2015:409245. doi: 10.1155/2015/409245 25977921 PMC4421034

[51] Jeon IS, Kim HR, Shin E, Kim EG, Han H, Hong J. Modulation of store-operated calcium entry and nascent adhesion by p21-activated kinase 1. Experimental & Molecular Medicine. 2018;50. 10.1038/s12276-018-0093-2 PMC5960643 29780159

[52] Lopez-Guerrero AM, Tomas-Martin P, Pascual-Caro C, Macartney T, Rojas-Fernandez A, Ball G, et al. Regulation of membrane ruffling by polarized STIM1 and ORAI1 in cortactin-rich domains. Sci Rep. 2017;7(1):383. doi: 10.1038/s41598-017-00331-4 28341841 PMC5428229

[53] Wang J-Y, Chen B-K, Wang Y-S, Tsai Y-T, Chen W-C, Chang W-C, et al. Involvement of store-operated calcium signaling in EGF-mediated COX-2 gene activation in cancer cells. Cell Signal. 2012;24(1):162–9. doi: 10.1016/j.cellsig.2011.08.017 21924350

[54] Emeriau N, de Clippele M, Gailly P, Tajeddine N. Store operated calcium entry is altered by the inhibition of receptors tyrosine kinase. Oncotarget. 2018;9(22):16059–73. doi: 10.18632/oncotarget.24685 29662626 PMC5882317

[55] Weidinger C, Shaw PJ, Feske S. STIM1 and STIM2-mediated Ca(2+) influx regulates antitumour immunity by CD8(+) T cells. EMBO Mol Med. 2013;5(9):1311–21. doi: 10.1002/emmm.201302989 23922331 PMC3799488

