# Supplementary material for: Context-dependent dysregulation of store-operated calcium channels in head and neck squamous cell carcinoma
Source: PLoS One. 2026 Mar 9;21(3):e0344393. doi: 10.1371/journal.pone.0344393 (PMC12970912; doi:10.1371/journal.pone.0344393)
Supplement: S1 File — (DOCX) [file pone.0344393.s001.docx]

**Supplementary Table S1. List of primers used for RT-qPCR**

| **Gene Name** | **Abbreviation** | **Species** | **Primer Sequence (5' → 3')** | **Amplicon size (bp)** |
| --- | --- | --- | --- | --- |
| ORAI-Calcium-Release-Activated Calcium Modulator-1 | ORAI1 | Human | F: GCCCTTCGGCCTGATCTTTAT  R: TGGAACTGTCGGTCAGTCTTAT- | 81 |
| ORAI-Calcium-Release-Activated Calcium Modulator-2 | ORAI2 | Human | F: CCTGTCGTGGCGGAAGCTCT  R: CGGGTACTGGTACTGCGTCT | 130 |
| ORAI-Calcium-Release-Activated Calcium Modulator-3 | ORAI3 | Human | F: GGCCAAGCTCAAAGCTTCC  R: CCTGGTGGGTACTCGTGGT | 105 |
| Stromal-Interaction Molecule-1 | STIM1 | Human | F: TGTGGAGCTGCCTCAGTATG  R: CTTCAGCACAGTCCCTGTCA | 109 |
| Stromal-Interaction Molecule-2 | STIM2 | Human | F: TTGGACCCTTGAAGACACTCT R: CCAGTTATGAGGTGGGCGTG | 238 |
| Glyceraldehyde-3-Phosphate Dehydrogenase | GAPDH | Human | F: TTGGCTACAGCAACAGGGTG R: GGGGAGATTCAGTGTGGTGG | 161 |

**Supplementary Table S2. Comprehensive primer validation for RT-qPCR experiments**

This table summarizes the in silico validation of all qPCR primer pairs used in this study. Primer sequences, predicted product sizes, and validation results from UCSC Genome Browser In-Silico PCR (genomic and transcript databases) and NCBI Primer-BLAST are shown. Experimental validation included melt curve analysis, no-reverse transcriptase (–RT) controls, and no-template controls (NTCs).

| Gene | Primer Sequences (5′→3′) | Primer Properties (F/R) | Amplicon Size (bp) | Primer-BLAST Targets (RefSeq NM_) | GENCODE Matches (ENST_) | Chromosome / Genomic Coordinates | Predicted Genomic Amplicon (bp) | Off-Target Hits | Experimental Validation |
| --- | --- | --- | --- | --- | --- | --- | --- | --- | --- |
| ORAI1 | F: GCCCTTCGGCCTGATCTTTAT R: TGGAACTGTCGGTCAGTCTTAT | Length: 21 / 22 bp Tm: 60.2 / 58.8 °C GC%: 52.4 / 45.5 | 81 | NM_032790.4 | ENST00000715685.1; ENST00000617316.2; ENST00000646827.1; ENST00000698901.2 | chr12:121,641,469-121,641,549  chr12:121641469+  121641549 | 81 | None detected | Single melt peak; –RT negative; NTC clean |
| ORAI2 | F: CCTGTCGTGGCGGAAGCTCT R: CGGGTACTGGTACTGCGTCT | Length: 20 / 20 bp Tm: 64.6 / 61.3 °C GC%: 65.0 / 60.0 | 130 | NM_032831.4  NM_001271818.2  NM_001126340.3 | ENST00000473939.2  ENST00000356387.6  ENST00000611770.5  ENST00000403646.8  ENST00000952428.1  ENST00000495936.7  ENST00000478730.7  ENST00000498661.6 | chr7:102,439,097–102,446,557 | 7,461 | TUBGCP2 (133 bp, 2–3 mismatches) | Large genomic size prevents gDNA amplification , single melt peak; –RT negative, NTC clean |
| ORAI3 | F: GGCCAAGCTCAAAGCTTCC R: CCTGGTGGGTACTCGTGGT | Length: 19 / 19 bp Tm: 59.4 / 60.9 °C GC%: 57.9 / 63.2 | 105 | NM_152288.3 | ENST00000318663.5; ENST00000566237.1; ENST00000563161.1 | chr16:30949463+  30953234 | 3,772 | ORAI1 (105 bp, 1–2 mismatches); ZSCAN20, MYO15A, LDLRAD4 (>3 mismatches) | Large genomic size prevents gDNA amplification; single melt peak; –RT negative, NTC clean |
| STIM1 | F: TGTGGAGCTGCCTCAGTATG R: CTTCAGCACAGTCCCTGTCA | Length: 20 / 20 bp Tm: 59.5 / 59.6 °C GC%: 55.0 / 55.0 | 109 | 17 RefSeq variants  (e.g.,  NM_001382567.1) | 24 total ENST IDs (e.g.,  ENST00000229239.10) | chr11:4055572+  4059323 | 3,752 | CEACAM1 (~2,100 bp,); SAR1B (945 bp, 2–3 mismatches) | Large genomic size prevents gDNA amplification; single melt peak; –RT negative, NTC clean |
| STIM2 | F: TTGGACCCTTGAAGACACTCT R: CCAGTTATGAGGTGGGCGTG | Length: 21 / 20 bp Tm: 58.6 / 60.7 °C GC%: 47.6 / 60.0 | 238 | NM_020860.4  NM_001169118.2  NM_001169117.2 | ENST00000494628.6  ENST00000465503.6  ENST00000463501.5  ENST00000467011.6  ENST00000698882.1  ENST00000467087.7  ENST00000477474.3 | No genomic match | None (exon-spanning) | None detected | Single melt peak; –RT negative; NTC clean |
| GAPDH | F: TTGGCTACAGCAACAGGGTG R: GGGGAGATTCAGTGTGGTGG | Length: 20 / 20 bp Tm: 60.5 / 60.0 °C GC%: 55.0 / 60.0 | 161 | NM_002046.7  NM_001256799.3  NM_001289745.3  NM_001289746.2  NM_001357943.2 | 32+ ENST IDs (e.g., ENST00000229239.10) | chr12:6,539,000–6,540,161 (1,161 bp) | 161 | ARHGEF11 (2,420 bp); CILP (3,384 bp, >3 mismatches) | Single melt peak; –RT negative; NTC clean |
