# Supplementary material for: Context-dependent dysregulation of store-operated calcium channels in head and neck squamous cell carcinoma
Source: PLoS One. 2026 Mar 9;21(3):e0344393. doi: 10.1371/journal.pone.0344393 (PMC12970912; doi:10.1371/journal.pone.0344393)
Supplement: S2 File — (DOCX) [file pone.0344393.s002.docx]

**
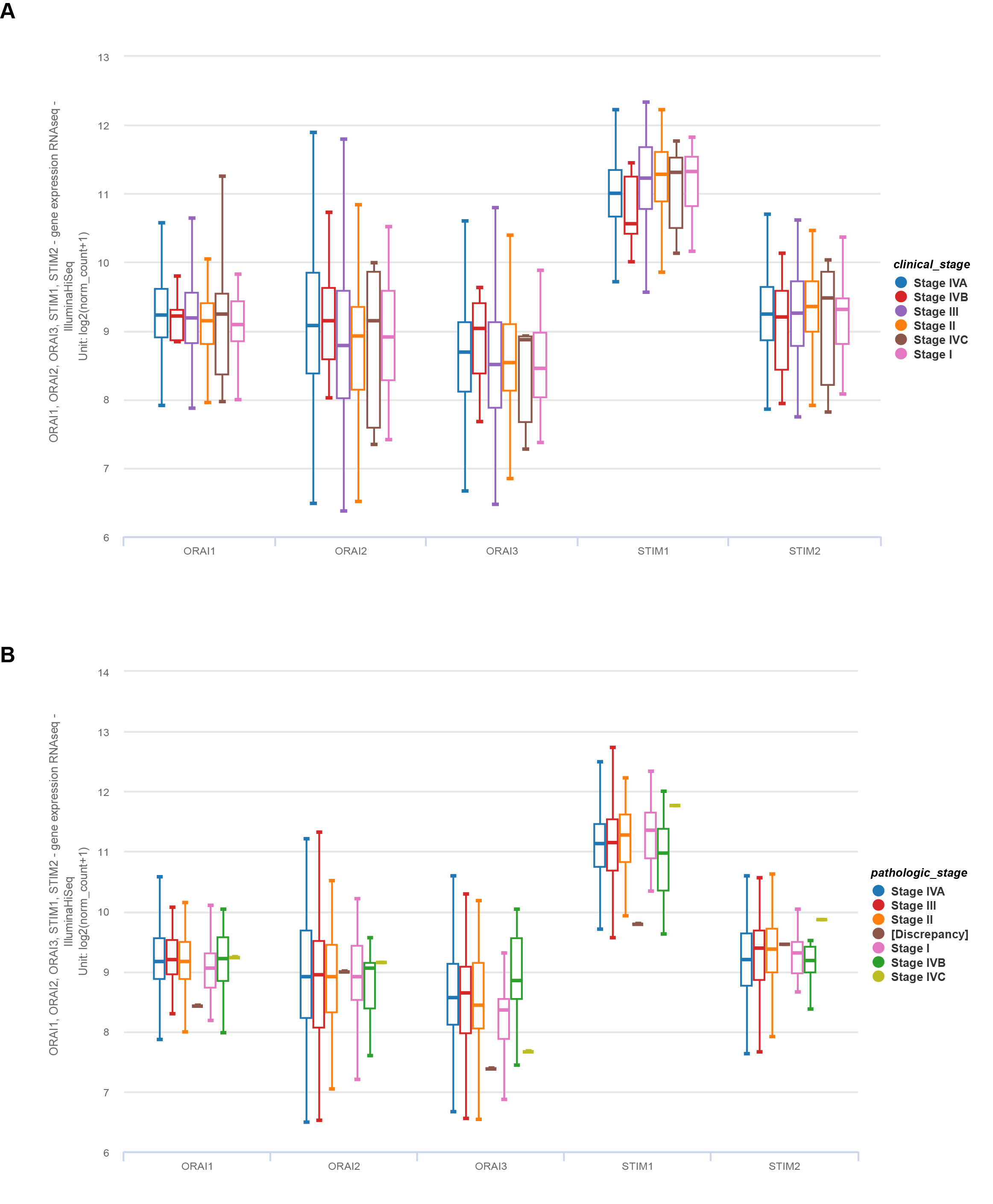
**

**Figure S1. Differential expression of ORAI and STIM isoforms by tumor stage in HNSCC.** (A) Clinical stage (I–IVB). (B) Pathological stage.

**
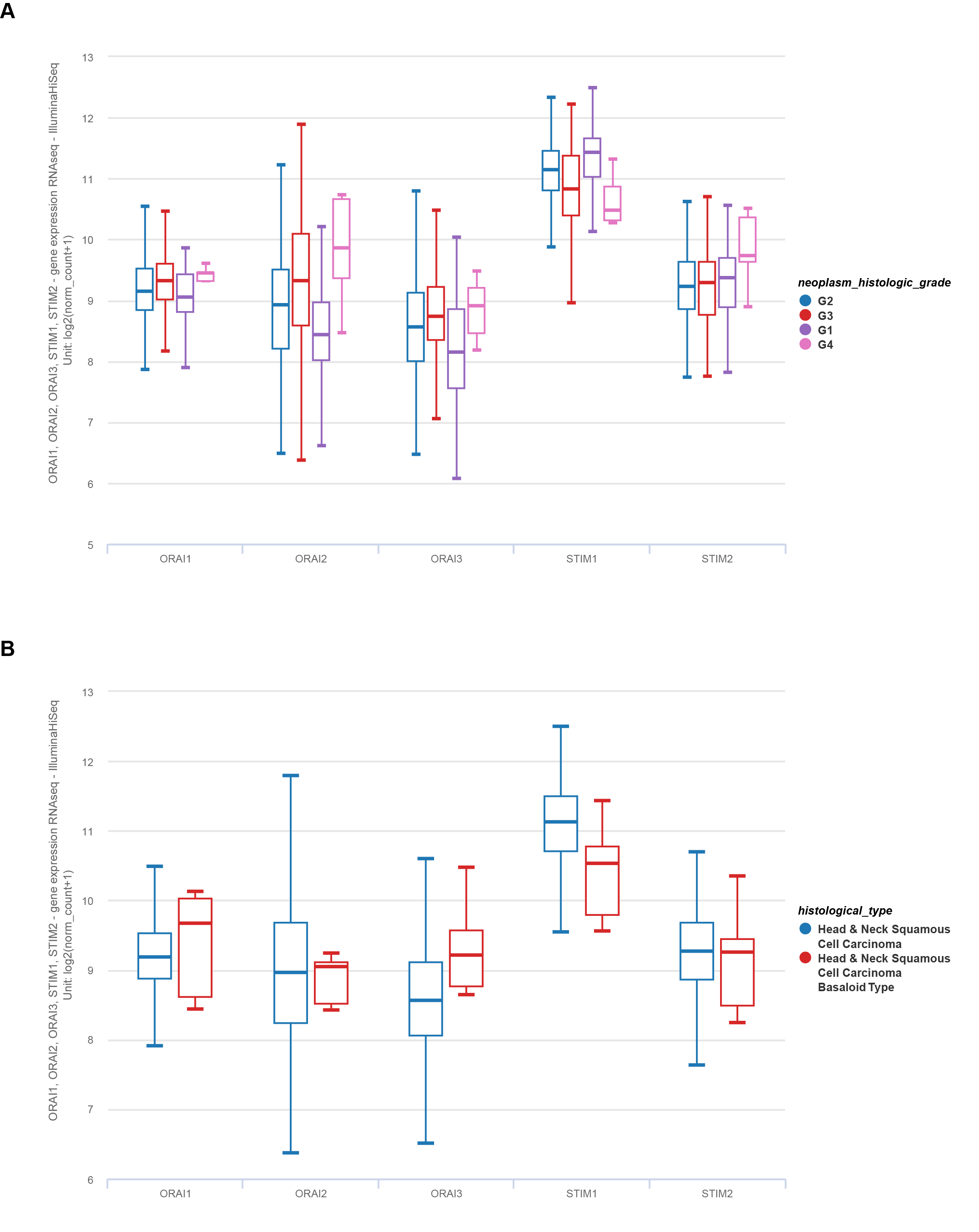
**

**Figure S2. Differential expression of ORAI and STIM isoforms in HNSCC histologic subtypes and grades.** (A) Tumor grade (G1–G4). (B) Histologic subtype (conventional vs. basaloid SCC)

**
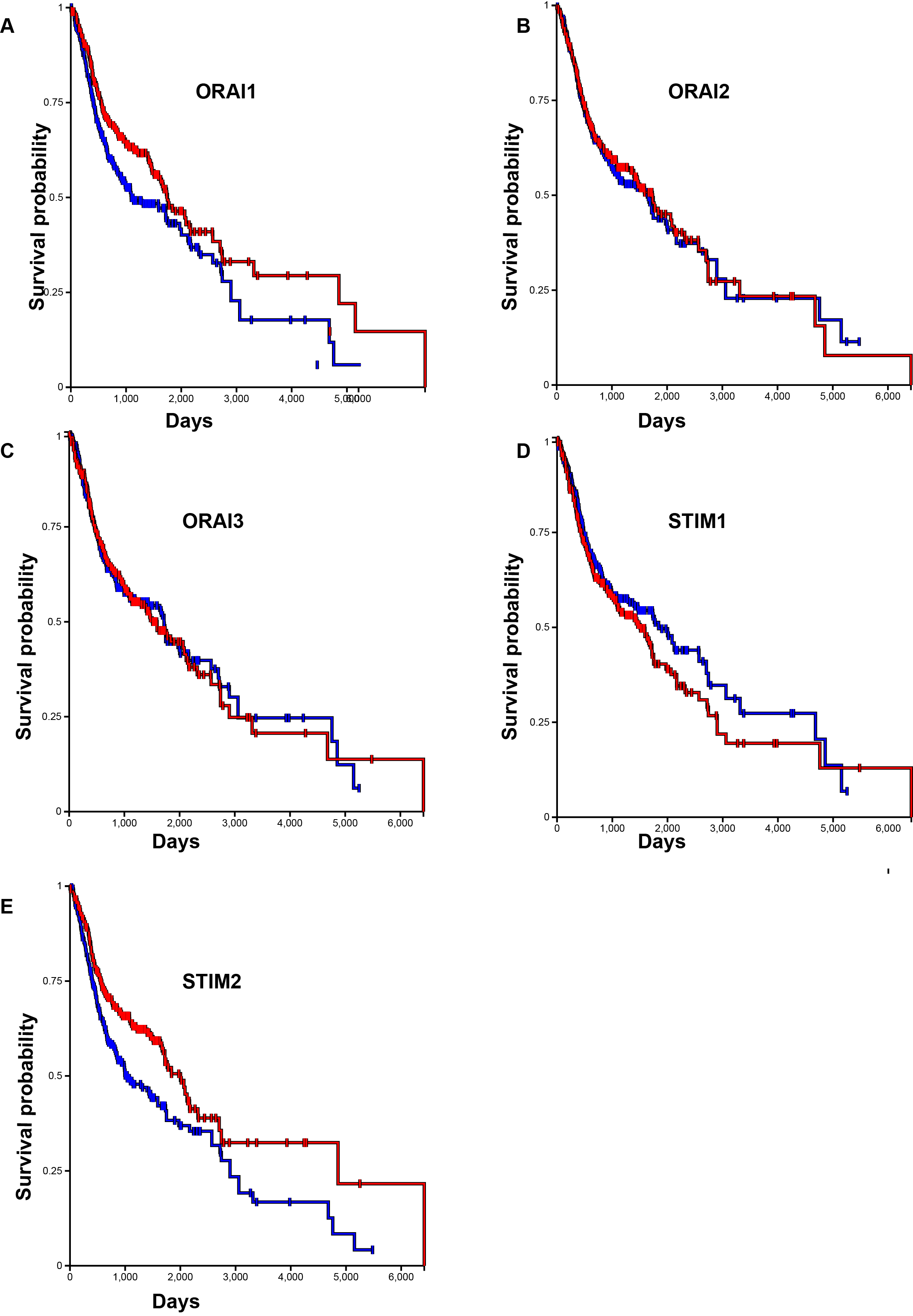
**

**Figure S3. Association between SOCE component expression and overall survival in the TCGA HNSCC cohort.** Kaplan-Meier curves for (A) ORAI1, (B) ORAI2, (C) ORAI3, (D) STIM1, and (E) STIM2.

**
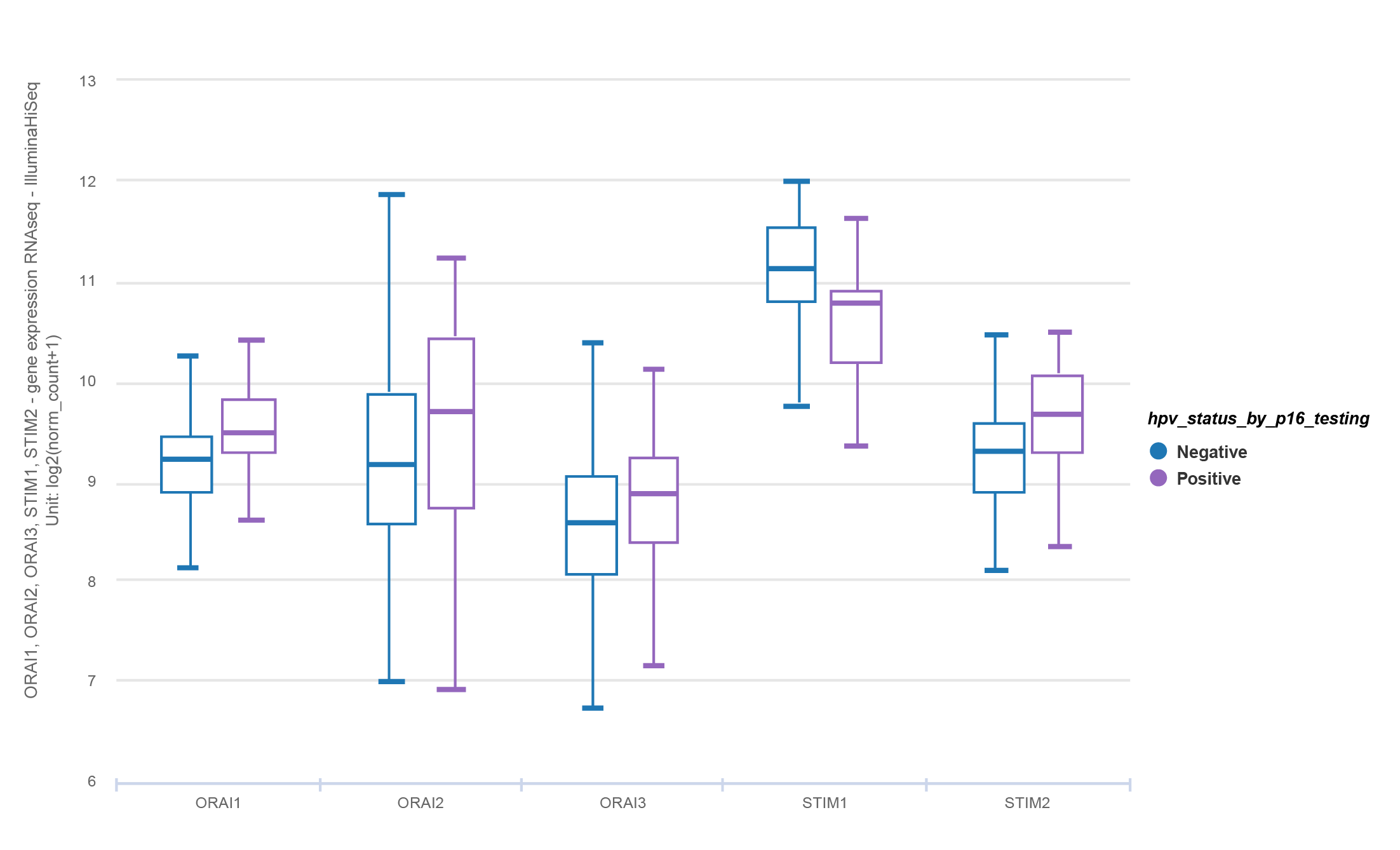
**

**Figure S4. SOCE isoform expression is shaped by HPV status.** SOCE component expression in HPV-positive vs. HPV-negative tumors.


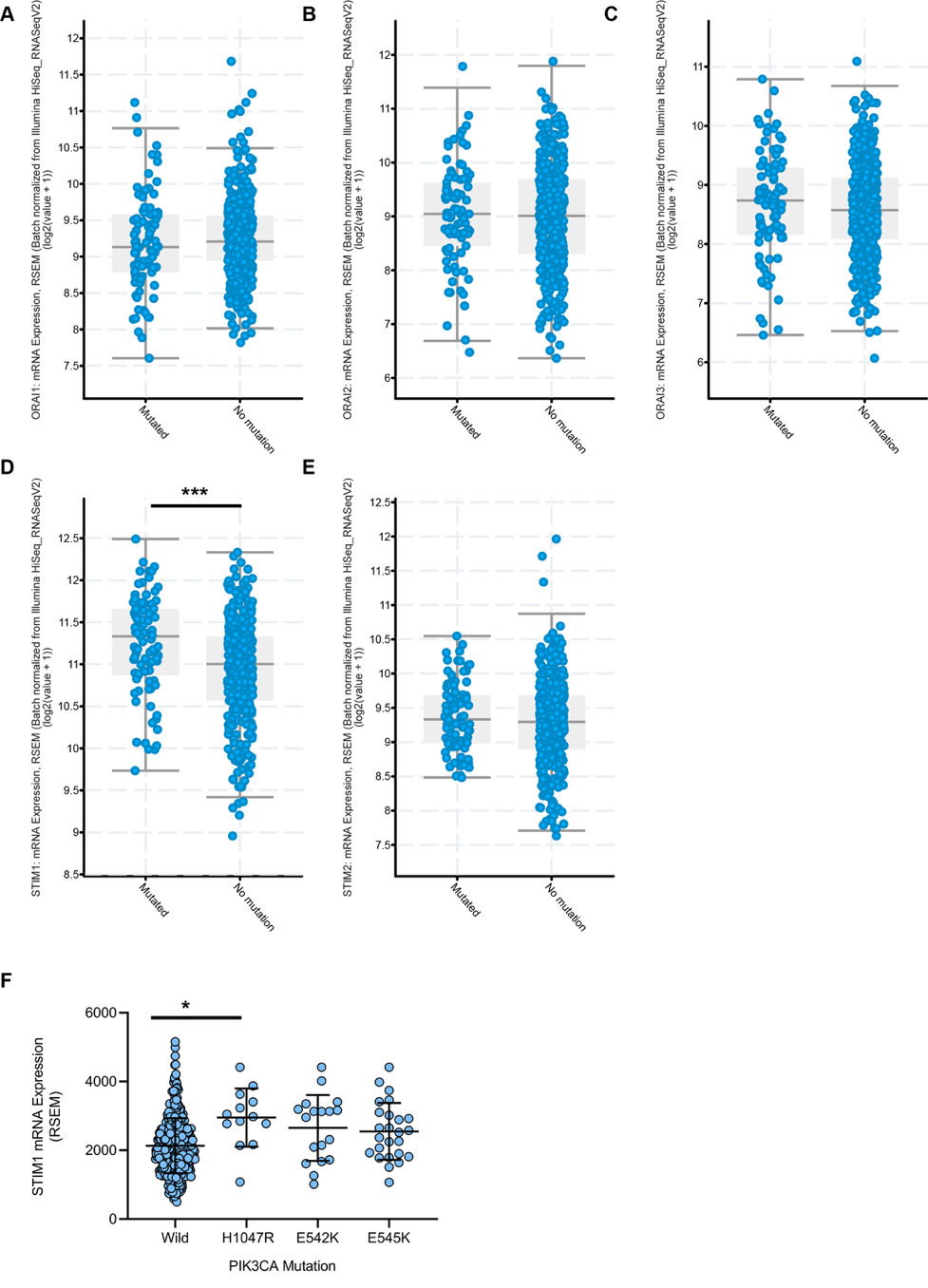


**Figure S5. Expression of SOCE components in relation to PIK3CA mutation status.**
Boxplots showing mRNA expression (log₂[RSEM+1], batch-normalized) of (A) ORAI1, (B) ORAI2, (C) ORAI3, (D) STIM1, and (E) STIM2 in PIK3CA wild-type (WT) versus PIK3CA-mutated tumors from the TCGA HNSCC cohort. Boxes indicate median and interquartile range (IQR), whiskers represent the full range, and individual tumors are shown as dots. Statistical comparisons for WT versus all PIK3CA-mutated tumors were performed using the Mann–Whitney test.
(F) Custom analysis of STIM1 expression stratified by PIK3CA mutation subtype (H1047R, E542K, and E545K). Boxplots show median and IQR with all individual values displayed. Statistical comparisons were performed using Welch’s t-test. **p* < 0.05, ****p* < 0.001.


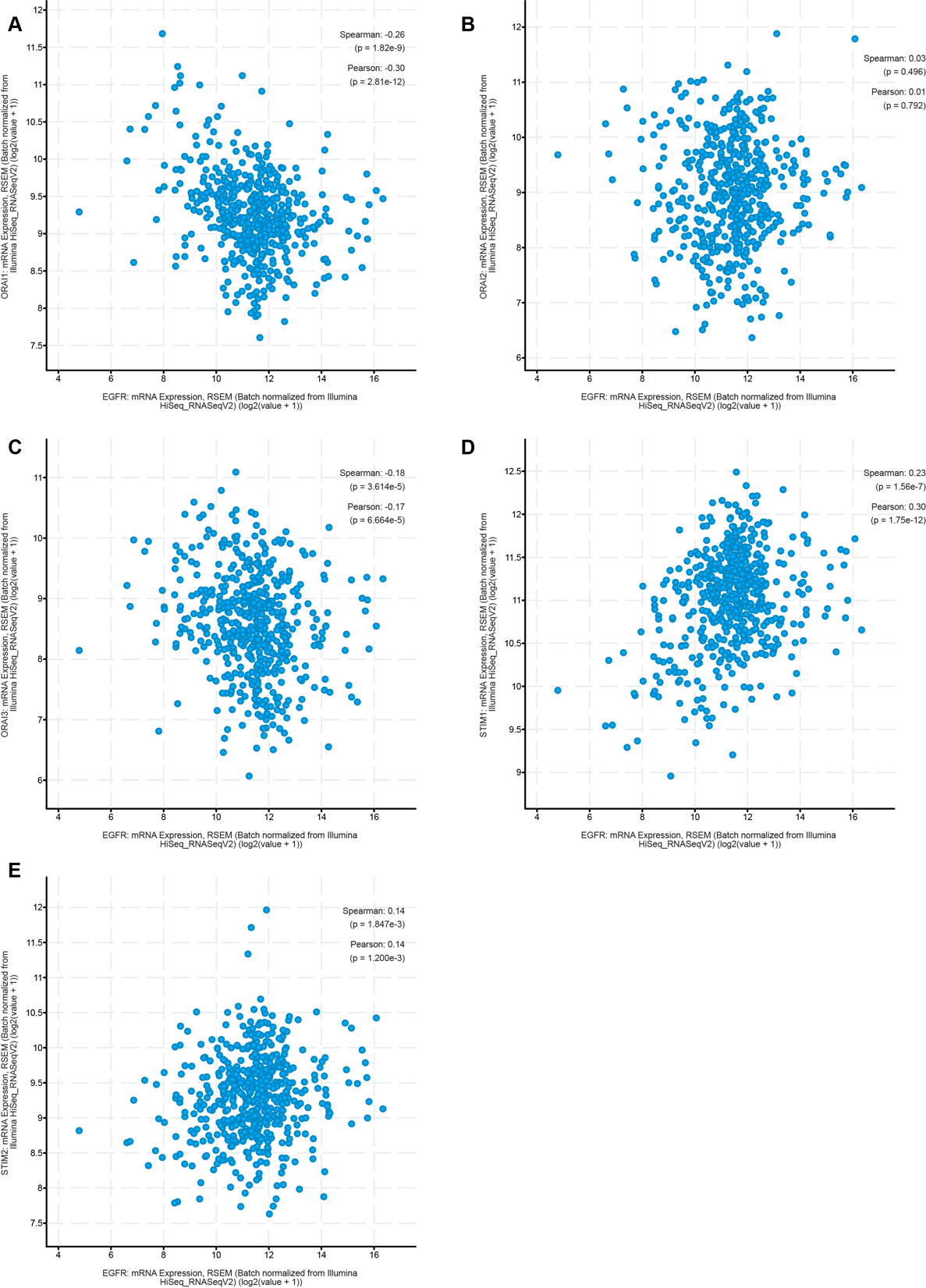


**Figure S6. Correlation of EGFR expression with SOCE components in HNSCC tumors.**

Scatterplots showing the relationship between EGFR mRNA expression and SOCE components in the TCGA HNSCC cohort: (A) ORAI1, (B) ORAI2, (C) ORAI3, (D) STIM1, and (E) STIM2. Spearman and Pearson correlation coefficients (r) and *p*-values are indicated on each plot.


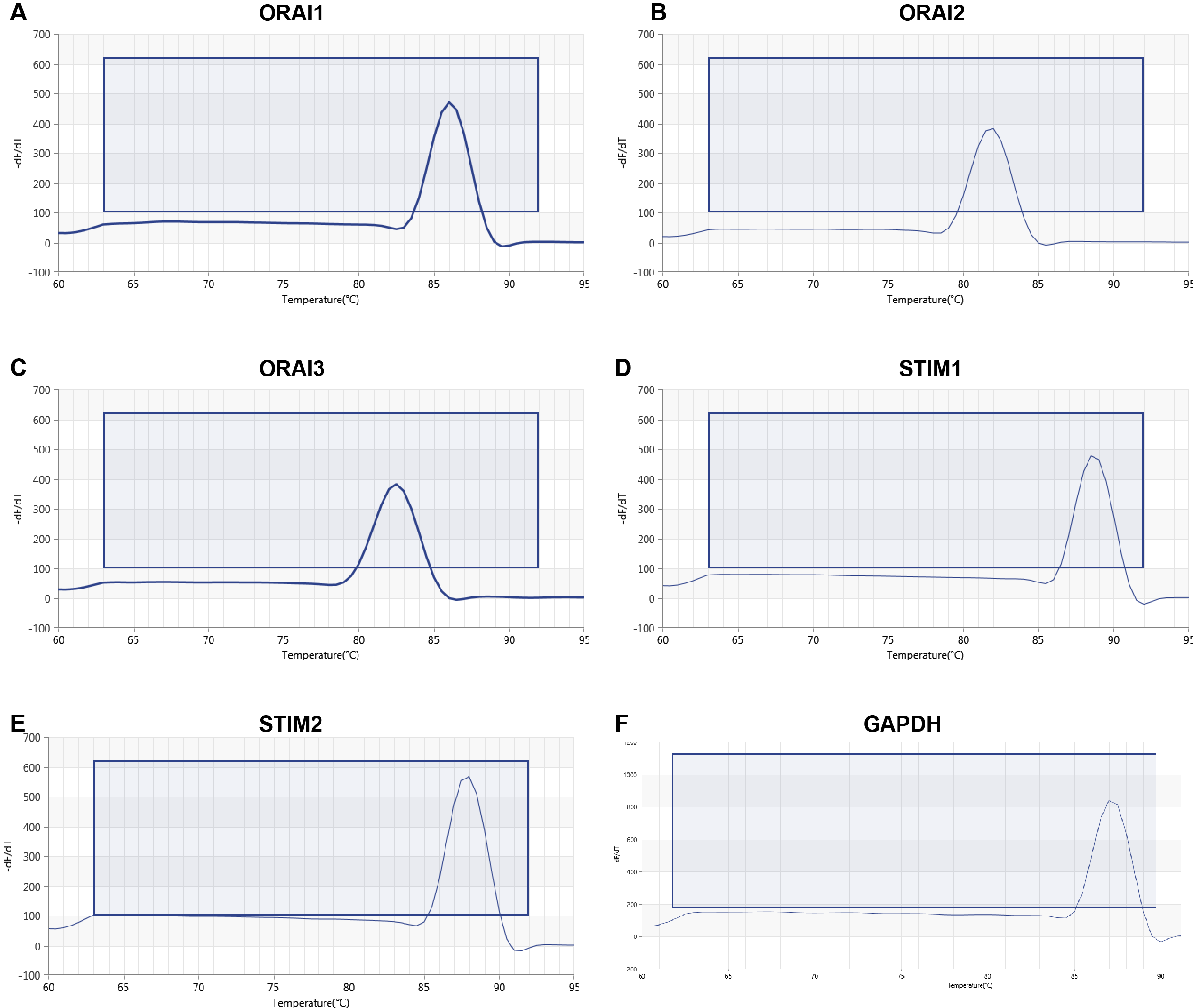


**Figure S7. Specificity validation by melting curve analysis:**
Dissociation (melt) curve analysis was performed following RT–qPCR amplification to verify primer specificity and exclude non-specific products or primer–dimer formation. Representative melt curves are shown for primers targeting (A) ORAI1, (B) ORAI2, (C) ORAI3, (D) STIM1, (E) STIM2, and (F) GAPDH. All primer pairs produced a single, sharp melting peak, consistent with amplification of a single specific amplicon. No secondary peaks were detected, indicating absence of non-specific amplification or primer–dimers.Melting curve profiles were consistent across independent biological replicates and experimental runs.
